# Dynamic imaging of cellular pH and redox homeostasis with a genetically encoded dual-functional biosensor, pHaROS, in yeast

**DOI:** 10.1074/jbc.RA119.007557

**Published:** 2019-09-05

**Authors:** Hang Zhao, Yu Zhang, Mingming Pan, Yichen Song, Ling Bai, Yuchen Miao, Yanqin Huang, Xiaohong Zhu, Chun-Peng Song

**Affiliations:** Department of Biology, Institute of Plant Stress Biology, State Key Laboratory of Cotton Biology, Henan University, Kaifeng, China 475001

**Keywords:** biosensor, organellar pH homeostasis, Saccharomyces cerevisiae, flavoprotein, redox signaling, fluorescent probe, iLOV, light, oxygen, or voltage sensing (LOV), mBeRFP

## Abstract

Intracellular pH and redox states are critical for multiple processes and partly determine cell behavior. Here, we developed a genetically encoded dual-function probe, named p H and redox-sensitive fluorescent protein (pHaROS), for simultaneous real-time detection of changes in redox potential and pH in living cells. pHaROS consists of the *Arabidopsis* flavin mononucleotide-binding fluorescent protein iLOV and an mKATE variant, mBeRFP. Using pHaROS in *Saccharomyces cerevisiae* cells, we confirmed that H_2_O_2_ raises the overall redox potential of the cell and found that this increase is accompanied by a decrease in cytosolic pH. Furthermore, we observed spatiotemporal pH and redox homeostasis within the nucleus at various stages of the cell cycle in budding yeast (*Saccharomyces cerevisiae*) during cellular development and responses to oxidative stress. Importantly, we could tailor pHaROS to specific applications, including measurements in different organelles and cell types and the GSH/GSSG ratio, highlighting pHaROS's high flexibility and versatility. In summary, we have developed pHaROS as a dual-function probe that can be used for simultaneously measuring cellular pH and redox potential, representing a very promising tool for determining the cross-talk between intracellular redox- and pH-signaling processes in yeast and mammalian U87 cell.

## Introduction

Cellular pH and redox homeostasis are critical for multiple processes that occur throughout the yeast, animal, and plant development (1–4). Dynamic changes in redox status and pH are interdependent processes, which is often ignored (5–7). For instance, the redox systems of the plasma membrane are involved in homeostasis of cytoplasm pH (8). Thus, a dual-functional probe for monitoring the changes in redox and pH simultaneously would be a powerful tool for revealing the relationship between redox status and pH. However, such a probe remains a challenge in terms of both design and validation.

Genetically encoded green fluorescent protein (GFP)-based sensors have been widely and successfully used to monitor dynamic changes in pH and redox status of living cells at the subcellular level (9–13). However, all these probes have been used separately and are unsuitable to be applied in combination due to the spectral overlap. More importantly, most GFP-based redox sensors like Hyper or roGFP probes, are affected, to some extent, by pH due to their structural characteristics (9, 14–16).

A new class of genetically encoded fluorescent proteins based on the light, oxygen, or voltage-sensing (LOV)^3^ flavin-binding domain holds substantial potential as fluorescent biosensors given its advantages over the current GFP reporters because of its pH and thermal stability as well small size (17–26). Recently, LOV-based proteins were exploited as metal or heavy metal sensors for measuring copper ion, mercury ion, and arsenic ion (19, 27, 28); and a *Bacillus subtilis* originated iLOV variant EcFbFP was combined with yellow fluorescent proteins to generate a Forster resonance energy transfer (FRET)-based probe, FluBO for oxygen detection in the *Escherichia coli* (*E. coli*) cell (29). In this study, we explored the potential use of *Arabidopsis thaliana* phototropin2 (phot2) LOV2 domain-based proteins for reporting cellular redox status. Of these LOV2 domain-based proteins, LOV(C426A) contains a point mutation that prevents the reversible photocycle featured by the LOV2 domain (17, 30–33), and iLOV is an improved version of LOV (C426A) that displayed doubly enhanced fluorescence compared with LOV(C426A) (17). Another iLOV-based mutant iLOV2.1 (LOV2.1) showed stronger resistance to photobleaching compared with LOV(C426A) (24). The pH tolerance of LOV proteins, together with the variable redox state of the flavin mononucleotide (FMN) chromophore, suggests that they could be developed as redox reporters (17, 33–34). In addition, LOV-based fluorescent proteins show particular promise as biosensors for the following reasons: (i) their fluorescence is oxygen-independent, facilitating their use in anaerobic environments (18); and (ii) they are also smaller in size, ranging from 110 to 140 amino acids, compared with bulkier GFP-based probes (∼240 amino acids), so their use in translational fusions should be less disruptive.

To decipher interdependent effects of redox and pH on cellular activity in living cells, we have developed for the first time a dual-function, genetically encoded probe for the simultaneous real-time detection of changes in redox and pH in living cells. This biosensor, named pHaROS (pH- and redox-sensitive fluorescent protein, or iLOV-mBeRFP), combines the iLOV domain and an mKATE variant, blue light-excited red fluorescent protein (mBeRFP) (35). The iLOV portion of pHaROS can reversibly gain an electron and thus display fluorescence intensity changes corresponding (36) to the redox status, whereas the mBeRFP portion is proven to be sensitive to a shift in pH. More importantly, we also developed two pHaROS variants, GRX1-pHaROS and pHaROS-red, which feature increased redox specificity and an expanded range of detectable pH, respectively. These novel probes generate quantitative data on both pH and redox status in living cells with high spatial and temporal resolution.

## Results

### iLOV protein is redox-sensitive and suitable for redox imaging

**Figure 1.**
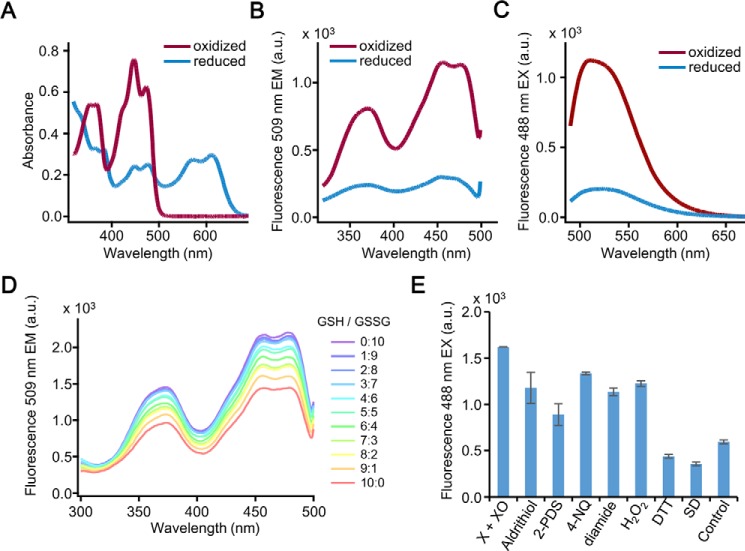
**Redox properties of iLOV protein *in vitro*.** *A,* absorbance spectrum of the iLOV protein upon exposure to H_2_O_2_ (2 mm) and DTT (50 mm) under UV excitation (280 nm). The purified iLOV protein from *E. coli* was incubated with H_2_O_2_ or DTT for 60 min and subjected to fluorescence analysis. The oxidized iLOV has an absorption peak at 450 nm and this absorption peak disappeared when iLOV was reduced with DTT. *B,* excitation spectrum of iLOV in oxidized and reduced states as described in *A. C,* emission spectrum of iLOV in oxidized and reduced states as described in *A. D,* titration analysis of the iLOV protein with the GSH/GSSG redox couple. The fluorescence of iLOV (1 μm) was measured in titration buffer (pH 7.5, 30 °C) containing different ratios of GSH/GSSG (10 mm total) corresponding to the designed redox potential (Eh) gradient. The excitation wavelength ranged from 300 to 500 nm. *a.u.*, arbitrary units. *E,* iLOV protein is sensitive to both oxidants and reductants. Reduced iLOV protein was treated with oxidants: 2 mm xanthine (*X*) and 20 units of xanthine oxidase (*XO*), 0.1 mm 4,4′-dithiodipyridine (*aldrithiol*), 0.1 mm 2,2′-dithiodipyridine (*2-PDS*), 0.1 mm 4-nitroquinoline N-oxide (*4-NQ*), 0.1 mm H_2_O_2_, 0.1 mm diamide; and reductants: 0.1 mm sodium dithionite (*SD*), 5 mm DTT.

The redox state of the FMN chromophore and its pH tolerance suggests that LOV proteins can be used as redox sensors and fluorescent reporters, even at variable pH (17, 32). Furthermore, it is well-established that cysteine mutants of LOV produce a flavin semiquinone upon reduction, and their fluorescence is resistant to photobleaching *in vitro* (17, 32). Consequently, we used two such *A. thaliana* LOV mutant proteins (iLOV (17, 18) and LOV2.1 (32)) to develop chimeras with mBeRFP in yeast, and tested their fluorescent properties. After expressing these two LOV2-based chimeric proteins in *E. coli*, we observed their fluorescence change upon treatment with 2 mm H_2_O_2_ or 5 mm dithiothreitol (DTT). Although H_2_O_2_ was able to significantly increase the fluorescence intensity of iLOV proteins, DTT treatment had the opposite effect and decreased the fluorescence (Fig. S1, *A* and *B*). LOV2.1 was brighter but less sensitive to redox agents compared with iLOV (Fig. S1, *C* and *D*). Surprisingly, we found that the absorption of iLOV protein differed significantly between the oxidized (treatment with H_2_O_2_, *red curve* in Fig. 1*A*) and the reduced state (treatment with DTT, *blue curve* in Fig. 1*A*). The fluorescent excitation and emission spectra were also recorded (Fig. 1, *B* and *C*). Upon exposure to H_2_O_2_, iLOV showed an excitation peak at 470 nm and an emission peak at 509 nm. By contrast, the fluorescence of iLOV protein was greatly reduced after the application of a sufficient amount of reductant such as DTT.

We next used the purified iLOV protein to investigate the variation of redox status upon exposure to titration buffers containing various ratios of reduced (GSH) and oxidized (GSSG) GSH (Fig. 1*D*). The fluorescence intensity of iLOV protein gradually increased with the increasing proportion of GSSG. The oxidation/reduction of iLOV by equilibration with GSH/GSSG was extremely fast. The midpoint redox potential of iLOV was −284.9 ± 2.0 mV, determined by applying the Nernst equation, with an *E*_0_ value of −240 mV for the GSH/GSSG redox couple at pH 7.0 (Fig. S2) (37). This value is close to the cytosolic redox potentials of yeast (37) suggesting sensitivity of iLOV toward cellular redox state. Similar results were obtained from submitting iLOV protein to other redox couples such as dihydrolipoate/lipoate, NAD^+^/NADH, and NADP^+^/NADPH (Fig. S3, *A–C*). iLOV also displayed sensitivity toward common oxidants (H_2_O_2_, O_2_^−^, diamide, etc.), as well as reducing agents, such as DTT (Fig. 1*E*). These data demonstrates that iLOV integrates the cellular redox potential into reporting its dynamics.

Based on these observations, iLOV protein was chosen for investigating improved *in vivo* imaging and easier observation of cellular redox state, and was expressed in yeast (*Saccharomyces cerevisiae* INVSc1). In these cells, iLOV showed relatively bright fluorescence (Fig. S3*D*), and, again, displayed increased fluorescence after the application of H_2_O_2_ (Fig. S3*E*).

Several experiments have shown that LOV proteins are very insensitive to pH variation (17). Thus, we detected iLOV fluorescence in various pH buffers *in vivo* and *in vitro* to evaluate whether it can indeed monitor redox changes in the cytosol without the interference of pH changes (Fig. S4, *A–C*). Fluorescence of iLOV protein was stable throughout the pH range of 5.4–9.0. Likewise, iLOV retained its fluorescence intensity in yeast cells when subjected to this series of pH values, following a digitonin pre-treatment that insures an instant equilibrium of the pH between the cytosol and the extracellular environment.

Furthermore, several divalent cations such as Ca^2+^ and Mg^2+^ were added to iLOV protein solutions at different concentrations to test the changes in its redox response. In these experiments, no significant alteration in fluorescence intensity (excitation and emission) was observed (Fig. S4, *D–G*), supporting previous reports (19). Although the fluorescence of iLOV protein has been reported to be sensitive to Cu^2+^ *in vitro* (19), we did not observe such sensitivity in yeast (Fig. S5, *A* and *B*), likely because of the greater cellular redox complexity of *in vivo* systems.

### mBeRFP protein is pH-sensitive and suitable for pH imaging

**Figure 2.**
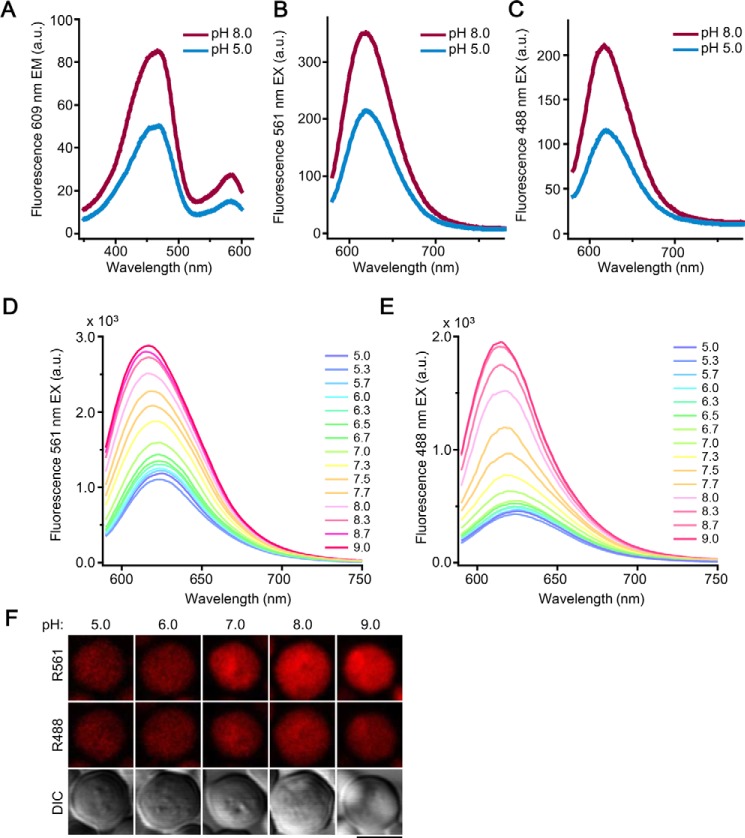
**Fluorescence of mBeRFP protein and its pH sensitivity *in vitro* and *in vivo*.** *A,* the excitation spectrum of mBeRFP protein in pH 5.0 and 8.0 buffers, detected at 609 nm. *B,* the emission spectrum of mBeRFP in pH 5.0 and 8.0 buffers (*I*_EX_ = 488 nm). *C,* the emission spectrum of mBeRFP in pH 5.0 and 8.0 buffers (*I*_EX_ = 561 nm). *D,* the fluorescence emission of mBeRFP in buffers of different pH recorded at 30 °C (*I*_EX_ = 561 nm). *E,* the fluorescence emission of mBeRFP in pH 5.0–9.0 buffers recorded at 30 °C (I_EX_ = 488 nm). *F,* yeast INVSc1 expressing mBeRFP was treated with pH 5.0–9.0 buffers; images were taken using emission wavelengths of 590 to 630 nm at excitation wavelengths of 488 and 561 nm, respectively. *Bar* = 5 μm.

The spectrum property of iLOV protein is similar to GFP. To avoid the spectrum overlap, we chose mBeRFP protein with a large Stokes shift, instead of GFP-based pH sensor as iLOV partner for creating a dual-functional biosensor. A previous study showed that mBeRFP has two excitation peaks, at 450 and 580 nm (35). Considering that the default configuration of a confocal laser scanning microscope includes 488 and 561 nm lasers, the emission intensity was determined at these excitation wavelengths. Our findings for mBeRFP under our experimental conditions are consistent with previous research, showing a large Stokes shift with the maximum excitation at both 450 and 580 nm, and emission at 609 nm (Fig. 2, *A* and *B*). Comparable with most GFP-based fluorescence proteins, we found mBeRFP fluorescence is sensitive to a pH change (Fig. 2, *A–C*), and can therefore be used as a pH probe. As shown by the spectrum in Fig. 2, *D* and *E*, the fluorescence intensity of mBeRFP protein at both excitation wavelengths gradually decreased, at different rates, with lowering pH, and the excitation peak reached its lowest point at a pH 5.0. mBeRFP protein showed no fluorescence intensity response to the concentration of either Ca^2+^ or Mg^2+^, with neither the excitation nor the emission peak being significantly affected (Fig. S6, *A–D*). In addition, mBeRFP fluorescence was stable in buffers with different concentrations of oxidants or reductants (Fig. S6*E*).

The results of the *in vivo* experiments were analogous to those obtained *in vitro* (Fig. 2*F*). Specifically, yeast expressing mBeRFP displayed a gradual increase in fluorescence intensity when excited at wavelengths of 488 and 561 nm. The rate of the increase was greater at 561 nm than at 488 nm (Fig. 2*F*). The fluorescence decrease in response to lowered pH was observed as well (Fig. 2*F*).

### Generation of dual-functional fluorescent probe pHaROS for detecting pH and redox homeostasis by ratio imaging

**Figure 3.**
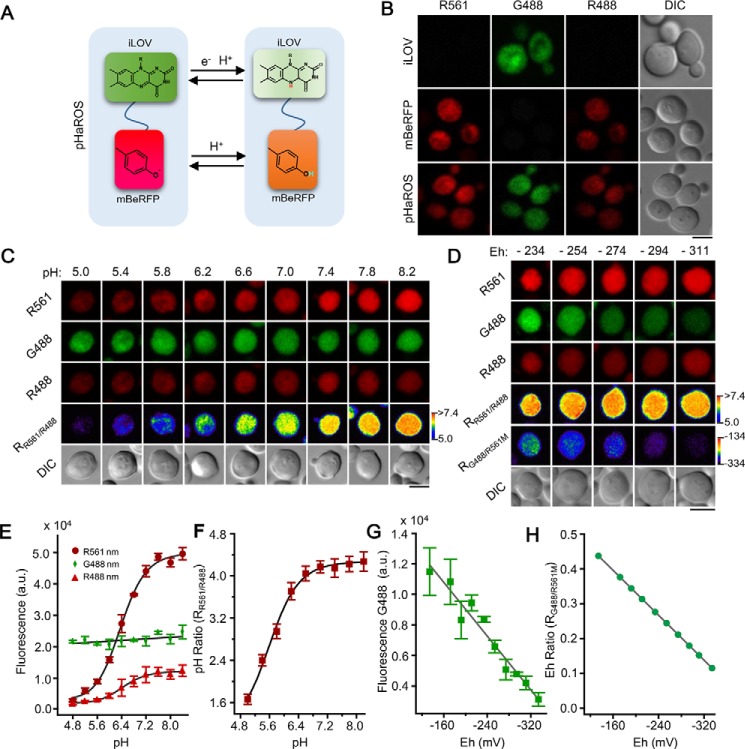
**pHaROS probe and the *in vivo* characteristics.** *A,* schematic model of pHaROS comprising iLOV and mBeRFP domains for sensing redox and pH homeostasis, respectively. *B,* comparison of fluorescence characteristics of pHaROS with those of iLOV and mBeRFP individually, in INVSc1 yeast cells. The emission wavelength has minimal spectral overlap. pHaROS can be excited at both 488 and 561 nm. iLOV, mBeRFP, and pHaROS proteins were excited at 488 and 561 nm wavelengths; fluorescence images were captured at emission wavelength ranges of 495–530 (*iLOV*) and 590–630 nm (*mBeRFP*). mBeRFP and iLOV at 488 nm are labeled as *R488* and *G488*, respectively, and mBeRFP at 561 nm is labeled as *R561. C,* confocal images of pHaROS probe in yeast (after digitonin treatment) in buffer at different pH values upon excitation at 488 and 561 nm. *D,* confocal images of yeast containing the pHaROS probe, indicating Eh of buffer changes from −234 to −311 mV. *E,* fluorescence intensity of pHaROS in yeast in pH 5.0–8.2 buffer. Yeast cells with pHaROS biosensor were treated with digitonin for 10 min before being incubated in buffers of different pH. Fluorescence intensity of iLOV upon excitation at 488 nm (Eh of the cytoplasm) reached a constant level because of digitonin treatment, but fluorescence intensity of mBeRFP upon excitation at 488 and 561 nm increased with rising pH. The mBeRFP fluorescence change fit with the Hill equation. *F, in vivo* calibration of pH ratio for pHaROS probe on excitation at 561 and 488 nm. The data were collected from ≥200 yeast cells and are presented as the mean ± S.D. (*n* = 3). *G,* iLOV fluorescence intensity of pHaROS probe in yeast in different Eh buffers (from −134 to −334 mV) upon excitation at 488 nm. The emission of iLOV fluorescence was recorded at 495–530 nm wavelengths. The data were collected from ≥200 yeast cells and are presented as the mean ± S.D. (*n* = 3). To generate different Eh values, reduced and oxidized DTT in various ratios (50 mm as final DTT concentration in pH 8.2 and 50 mm Tris-HCl buffer) were added. The experimental conditions were identical to those described for *C. H, in vivo* redox ratio calibration of pHaROS using G488/R561 max (R_G488/R561M_), whereby R561 max values were calculated as described in *C*, and the fluorescence of R561 will reach its maximum value when pH ≥ 8.2. *Bar* = 5 μm; pH and Eh levels have been pseudocolor coded according to the scale on the *right*.

The pHaROS chimeras and their mechanism for sensing pH and redox status used in the present study were shown schematically in Fig. 3*A*. The pH- and redox-sensitive biosensor construct encodes the complete iLOV and mBeRFP proteins; it also contains a short linker, GSTSNGRQCAGIL, which is inserted into the pHaROS protein between mBeRFP and iLOV. The fluorescence lifetime of this iLOV-mBeRFP fusion protein was measured to be almost the same as that of the parent iLOV protein, suggesting that there is no FRET between iLOV and mBeRFP (Fig. S7). These data also suggested that the linker between iLOV and mBeRFP did not affect the biosensor. When iLOV, mBeRFP, and iLOV-mBeRFP chimera were expressed in *S. cerevisiae*, fluorescence of each individual fluorescent protein and the fusion protein were observed upon excitation at 488 and 561 nm (Fig. 3*B*).

Next, we decided to check whether the individual functions of iLOV and mBeRFP were retained by pHaROS. To this end, fluorescence images of transgenic yeast cells expressing pHaROS were captured after the cells had been pretreated with 0.1% digitonin and transferred into different pH buffers for 10 min. As shown in Fig. 3*C*, mBeRFP fluorescence gradually increased with the rising pH, as did the ratio of intensities at 561 to 488 nm. As expected, the iLOV fluorescence was not affected by changing the pH of the incubation medium. We quantified the fluorescence signals of 200 or more yeast cells using ImageJ, and the correlation curve indicated that there is a clear difference in the 561/488 nm ratios between pH 5.2 and 6.8, but the curve reaches a plateau at pH > 7.2 (Fig. 3, *C*, *E*, and *F*).

At the same time, the *in vivo* correlation between cytosolic Eh (electric potential) and iLOV fluorescence was established using the same yeast as mentioned above, but with a different treatment. Although redox agents such as DTT can move freely through the cell membrane, it was still important to confirm that the iLOV protein is not affected by changes in pH. Thus, 0.1% digitonin and a pH 8.2 buffer were used to ensure the maintenance of yeast in an environment with a constant pH in yeast cells, and DTT was used to generate a series of Eh buffers that were calibrated using an ORP probe (Oxidation reduction potential probe, Thermo Orion Star). As shown in Fig. 3, *D* and *G*, the iLOV fluorescence intensity was linearly correlated with Eh in the range of −134 to −334 mV. The fluorescence was barely detectable at cytosolic Eh of −311 mV, and the fluorescence intensity increased as a cell became more oxidized. Using mBeRFP fluorescence intensity for normalization, a linear correlation was generated between the ratio of iLOV 488 nm (G488)/the maximum of mBeRFP 561 nm (R561M), and Eh at the range between −134 and −334 mV (for details of the calculation, see “Experimental procedures”) (Fig. 3, *G–H*).

The Eh of a reagent is known to be related to the pH (6, 7), and thus we recorded the changes in Eh of 50 mm DTT buffer that was adjusted to different pH values. Specifically, a lower pH led to a higher level of DTT-induced Eh. When yeast was incubated with DTT at a constant concentration but in buffers of different pH, the fluorescence levels of mBeRFP and iLOV exhibited independent changes. Specifically, iLOV fluorescence decreased with a decline of Eh, whereas mBeRFP fluorescence increased with rising pH (Fig. S8). This indicated that mBeRFP and iLOV act separately but simultaneously as pH and redox sensors, respectively.

### pHaROS and its variants for versatile applications in different cell types

**Figure 4.**
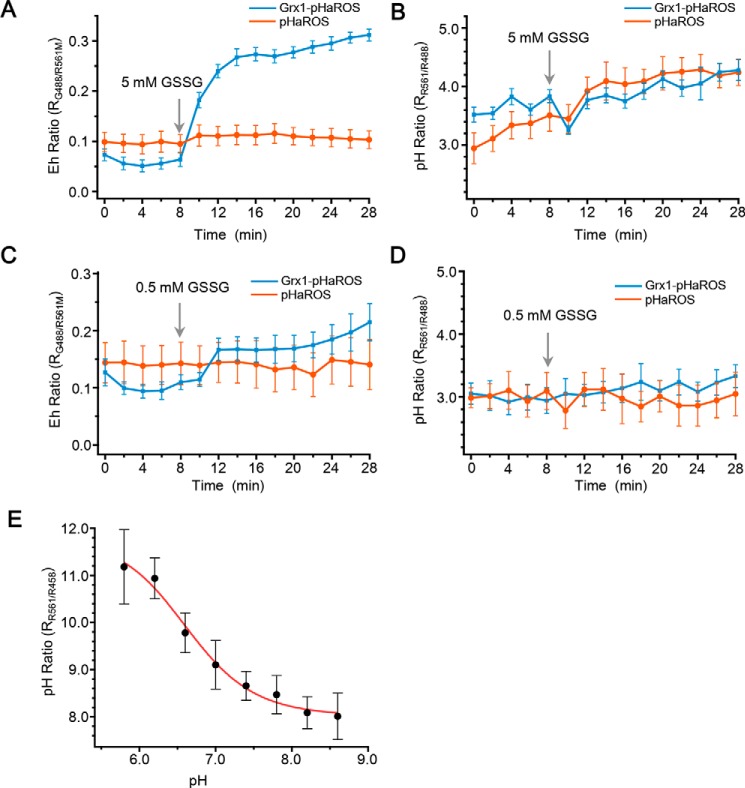
**Characterization of tailored pHaROS variants.** *A,* Eh ratio of GRX1-pHaROS increases rapidly, whereas the fluorescence ratio of pHaROS remains unchanged after adding 5 mm GSSG. *B,* pH ratios of GRX1-pHaROS and pHaROS change in a similar trend after 5 mm GSSG application. *C,* Eh ratio of GRX1-pHaROS increases gradually, whereas the fluorescence ratio of pHaROS remains unchanged after adding 0.5 mm GSSG. *D,* pH ratio of GRX1-pHaROS and pHaROS change in a similar trend after adding 0.5 mm GSSG. Data of *A–D* were collected from >25 individual yeast cells. *E,* pH ratio of pHaROS-red changes in response to the pH values of incubation buffer (5.8–8.6). Data were collected from ∼100 individual yeast cells.

To test whether pHaROS could be further optimized for desired specificity, in a similar way to what has been shown for Grx1-roGFP2 for reporting the GSH/GSSG ratio (38), or roGFP2-Orp1 probe for measuring H_2_O_2_ (39), to extend its great utility to particular signaling pathways, we constructed another pHaROS variant that contains a GRX1 protein at its N-terminal and named it GRX1-pHaROS (Fig. S9, *A* and *B*). The fluorescence excitation spectra of iLOV were readily detectable in yeast cells expressing GRX1-pHaROS, and altered by the addition of GSSG (Fig. S9*B* and Fig. 4*A*). Compared with pHaROS, GRX1-pHaROS showed significantly increased sensitivity to GSSG (Fig. 4, *A* and *C*), whereas the pH sensitivity was not affected (Fig. 4, *B* and *D*).

Given that mBeRFP measured cellular pH in the acidic-to-neutral range, pHaROS-red, containing another red fluorescent version of the pH biosensor, was developed for enabling the detection of alkaline pH (Fig. S9, *C* and *E*). pHaROS-red was designed simply by replacing mBeRFP of pHaROS with pHred that has been used previously as the fluorescent pH sensor (40). The pHaROS-red is extremely sensitive to physiological pH changes, showing a functional correlation between the 561/458 nm fluorescence ratio and the pH in the range of pH 5.8–8.6 in yeast cells (Fig. S9*D* and Fig. 4*E*).

To check if pHaROS can also be used in mammalian cells, we expressed pHaROS in U87 cells (U87, a human primary glioblastoma cell line) (41). A significant redox change was observed in U87 cells when applied with 100 μm H_2_O_2_ (Fig. S10), demonstrating that the pHaROS probe could serve as a versatile tool in different systems.

### The orchestration of redox and pH homeostasis in yeast monitored using pHaROS biosensor

**Figure 5.**
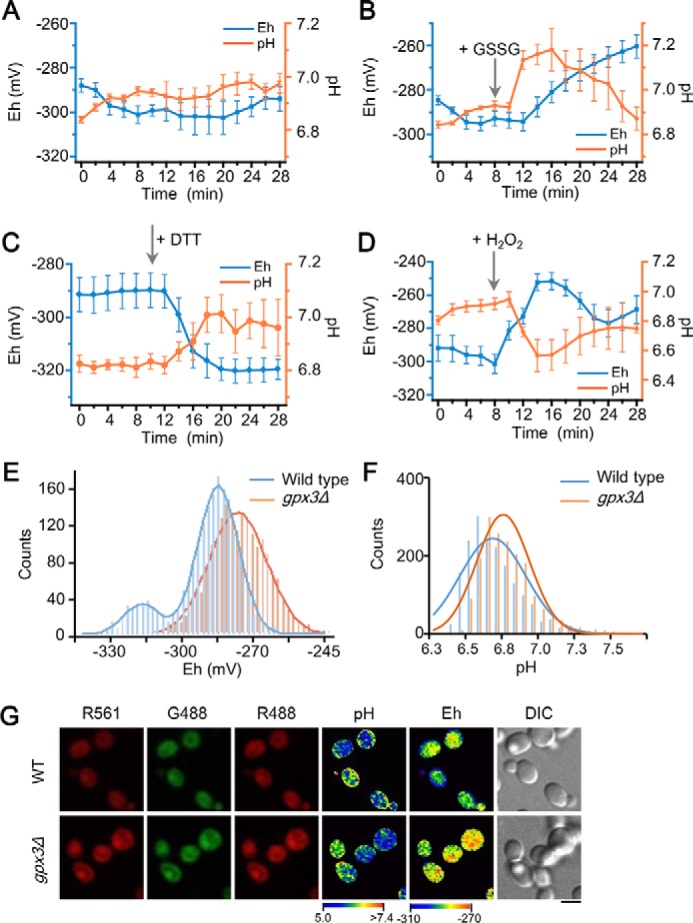
**pHaROS reveals the effect of redox agents on yeast cells and characterizes the cellular pH and redox state of WT *versus* H_2_O_2_–sensitized yeast.** *A,* the time course of the cytosolic pH as well as Eh was analyzed in yeast BY4741 cells expressing pHaROS, the fluorescence of which was monitored at different time points as indicated. *Blue lines* in *A–D* indicate the cytosolic Eh, whereas the *orange lines* indicate the cytosolic pH of WT yeast. *B,* cytosolic Eh and pH of yeast cells changed significantly upon the addition of 2 mm GSSG after 8 min of incubation with culture solution. *C,* cytosolic Eh of yeast cells dropped after exposure to 2 mm DTT, whereas the pH increased less significantly than upon treatment with GSSG. *D,* cytosolic Eh of yeast cells increased after the addition of 2 mm H_2_O_2_ and the pH dropped at the same time. The data in *A–D* were collected using >200 yeast cells and are presented as the mean ± S.D. (*n* = 3). *E,* the distribution of redox potential in WT and *gpx3*Δ cell populations; Eh of *gpx3*Δ cells is higher than that of WT cells. *F,* distribution of pH ratios in WT and *gpx3*Δ cell populations showed no significant difference. *G,* representative cell fluorescence, pH and Eh images of WT and *gpx3*Δ yeasts. The data in *E* and *F* were collected using 2500 yeast cells; *bar* = 5 μm; All data were captured with a spinning disc confocal microscope.

The ratio images of redox state and pH in yeast cells varied slightly over time: pH continued to slowly increase and redox potential initially decreased and then was maintained at a stable level, as reflected by the ratio images of pH and redox in pHaROS-expressing cells (Fig. 5*A*). We treated yeast with H_2_O_2_, DTT, and GSSG, and recorded the dynamic changes of pH and redox condition over time (Fig. 5, *B–D*). These three redox agents displayed three different patterns of change in redox and pH. Upon application of 2 mm GSSG (Fig. 5*B*), the redox potential increased after a 4-min lag, whereas pH exhibited a slight increase, followed by a gradual decline. Rebuilding equilibrium in cytosolic GSH/GSSH after the application of external GSSG consumes H^+^, resulting in alkalization of cytosol. In contrast, a dramatic decrease in redox potential was observed upon addition of 2 mm DTT, but only a slight change in cytosolic pH was observed in pHaROS-expressing cells (Fig. 5*C*). A significant increase in cytosolic redox potential, accompanied by a rapid decreased in pH, was observed due to treatment with 2 mm H_2_O_2_ (Fig. 5*D*). This might be due to the weak acid nature of H_2_O_2_. An alternative pH sensor, SNARF-1, confirmed the observed different directions of changes in pH upon application of H_2_O_2_ and GSSG (Fig. S11, *A* and *B*).

A lack of GSH peroxidase 3 (*gpx3*), which is involved in scavenging oxygen radicals in yeast, results in a lower tolerance to H_2_O_2_ (42, 43). The cell population of BY4741 and *gpx3*Δ strains differed both in cell size and *in vivo* redox potential despite being cultured under the same conditions. Wildtype (WT) cells were larger than *gpx3*Δ cells (Fig. S12*A*), whereas *gpx3*Δ mutants had a higher cytosolic redox potential than the WT cells (Fig. 5, *E* and *G*). The pH, on the other hand, was identical in the two strains (Fig. 5, *F* and *G*). When *gpx3*Δ mutants were treated with 0.2 mm DTT, the cell size increased compared with the untreated cells (Fig. S12*B*). By contrast, application of H_2_O_2_ results in the slower growth in the WT yeast (Fig. S12*C*). Therefore, the ability to scavenge ROS is reduced in the *gpx3*Δ population, which may lead to smaller cell size under normal growth conditions.

### The oscillation of nuclear pH and Eh during the yeast cell cycle

**Figure 6.**
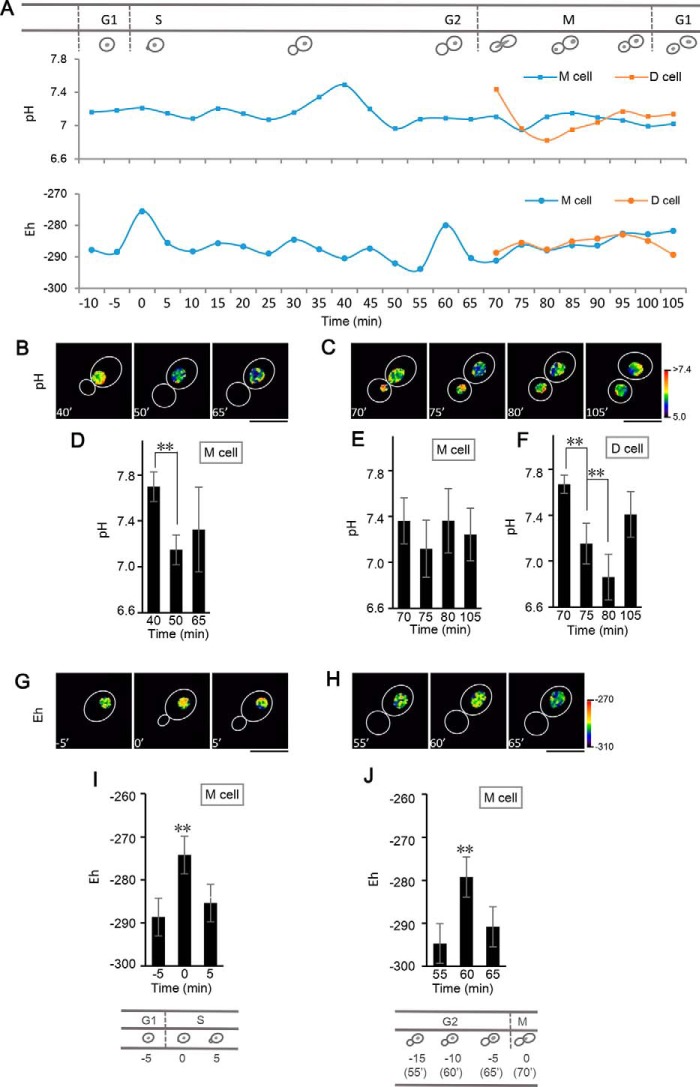
**pHaROS enables monitoring of the changes in pH and redox potential in the nucleus of budding yeast.** *A,* time course of nuclear pH and redox state in yeast cells (BY4741) expressing NLS-pHaROS (*blue line*, a representative mother cell; *orange line*, a representative daughter cell), the fluorescence of which was monitored during budding. *Top panel*, schematic diagram of the cell cycle during budding; *middle panel*, pH oscillation during the cell cycle; *bottom panel*, Eh oscillation during the cell cycle. *M cell*, mother cell; *D cell*, daughter cell. *B* and *C,* representative pseudo-colored ratio images of pH distribution in the nucleus at start points (*B*) and during the beginning of M phase (*C*) (Video S1). *D,* statistical data indicate the pH change in mother cell during the later S-G2 phases. *E* and *F,* pH oscillation of mother nucleus and daughter nucleus in the beginning of M phase. *G* and *H,* representative pseudo-colored ratio images of Eh distribution in the nucleus at the start point of budding (*G*) and before the M phase (*H*) (Video S2). *I* and *J,* Eh peak of mother cell during G1-S transition (*I*) and before M phases (*J*). Because the times of the cell cycle are variable due to cell states, the statistical data were selected from the yeast cells, which experienced the same time lapse during cell cycle and the cells with 105 min budding length as displayed in our model. *D*, *F*, *I*, and *J,* **, *p* < 0.01). *Bars* indicate the mean ± S.D. (*n* ≥ 30 yeast cells). *Bar* = 5 μm.

Numerous studies have indicated that eukaryotic cells display the oscillation of intracellular pH and redox state through their cell cycle due to a series of events such as DNA decompaction and synthesis, and RNA, protein, and lipid synthesis (44). However, to our knowledge, the changes in nuclear pH and redox condition during the cell cycle are not known. We addressed this question by performing pH and redox ratio imaging analysis of the BY4741 yeast strain during budding using the nucleus-localizing probe, NLS-pHaROS. Fig. 6*A* shows that the pH and redox status in the nucleus varies temporally during budding. In mother cells, a weak oscillation of the nuclear pH occurs in the S phase (Fig. 6*A*), whereas it is then sustained at a steady state level in the G2 phase. The pH appears to peak in the nucleus of the mother cell during the S-G2 phases, about 30 min before the M phase (Fig. 6, *B* and *D*). In daughter cells, a transient alkalization occurs during the early G1 phase following nucleus migration, but then the nucleus gradually acidifies to ∼pH 6.81. On the other hand, the nuclear pH in mother cells did not experience the same changes as that in daughter cells (Fig. 6, *A* and *C*, *E* and *F*). Two peaks of oxidation status occur, one at the start of S phase, and the other about 10 min before nuclear migration (Fig. 6, *A* and *G–J*). During yeast budding in normal conditions, we observed an interesting phenomenon in BY4741 yeast, *i.e.* incomplete budding process companied with abnormal redox or pH status (Fig. S13, Video S3–S8). It still needs to be investigated whether or not abnormal redox and pH status cause the failure of passing through cell cycle checkpoint.

It is known that GSH levels in nuclei play an important role in the cell cycle (45). *GLR1* encodes a cytosolic and mitochondrial GSH-disulfide reductase (Glr1). A defective allele of *GLR1* (*glr1*Δ) accumulates high levels of GSSG in yeast cells (46, 47). Moreover, it has been demonstrated that nuclear GSH levels are determined by the cytoplasmic GSH pool (48–50). To develop the specific redox probe for monitoring nuclear GSH/GSSG redox potential during the yeast cell cycle, the Grx1-fused pHaROS was expressed in the nuclei of BY4741 and *glr1*Δ mutant cells. As shown in Fig. S14, we clearly observed the variation of GSH/GSSG redox status during yeast budding. Similar to overall oxidation status, there are two peaks of G488/R561M at the start point of S phase and before the M phase, reflecting a drop of GSH/GSSG in these two phases (Fig. S14, *E–H*). Comparative to WT (BY4741), *glr1*Δ mutant cells show a lower GSH/GSSG redox state due to the accumulation of GSSG. To verify the GRX1-pHaROS sensitivity to GSH/GSSG, we also expressed the validated specific probe of GSH/GSSG (Grx1-roGFP2) in BY4741 and *glr1*Δ mutant cells. The Grx1-roGFP2 produces a similar variation pattern of GSH/GSSG examined by GRX1-pHaROS probe in both BY4741 and *glr1*Δ mutant cells (Fig. S14, *I–L*). These results indicate that NLS-Grx1-pHaROS, similar to the NLS-Grx1-roGFP2 probe, can detect specific changes in GSH/GSSG redox potential during the cell cycle.

## Discussion

Here, we report that the pHaROS probe can serve as a dual-functional indicator for the simultaneous visualization of intracellular pH and redox homeostasis in yeast. The chimeric protein pHaROS has two constituents: the small iLOV protein, which is insensitive to pH fluctuations but displays a reversible change in fluorescence signal when the cytosolic redox state fluctuates between −134 and −334 mV, and mBeRFP, a fluorescent protein, which is sensitive to pH in the range 5.2 to 6.8. As demonstrated in this study, pHaROS is a very effective fluorescent reporter because the short iLOV protein retains its spectral properties when fused to mBeRFP, whereas the large Stokes shift intrinsic to mBeRFP ensures that there is no spectral overlap or fluorescent resonance energy transfer when it is joined to iLOV. Upon excitation at 488 nm, mBeRFP and iLOV emit fluorescence signals at 609 and 512 nm, respectively. More importantly, mBeRFP has another excitation peak at 580 nm, which could be used for ratiometric calculations when monitoring pH changes in cells. Therefore, pHaROS allows the detection of dynamic changes in redox potential and pH at the same time.

pHaROS offers several improvements over the existing GFP-based reporter systems. First, unlike the GFP sensors, pHaROS can be reversibly photobleached, reaching full recovery within few minutes of complete bleaching under continuous laser scanning. Second, the fluorescence of iLOV within the pHaROS probe is not influenced by pH. For instance, the iLOV component of pHaROS is not only resistant to a pH range of 4 to 11, but also exhibits thermal stability (up to 60 °C) and rapid maturation of fluorescence (within 3 min) (36, 51). Third, iLOV responds quickly to electron exchange and can therefore detect transient changes in cytosolic redox potentials. Finally, iLOV and GFP feature similar spectral characteristics, and therefore iLOV is suitable for the wide range of fluorescent instruments that have been applied to GFP in the past.

In real-time, the iLOV domain within pHaROS is able to monitor redox homeostasis as a consequence of protonation (H^+^ and *e*^−^) of FMN (Fig. 3*A*). However, the redox homeostasis in the cell is co-determined by other diverse processes such as metabolism, energy production, apoptosis, disease, oxidative defense mechanisms, and various signal transduction pathways. For example, the cytosolic, chloroplastic, and mitochondrial thioredoxins are reduced by NADPH thioredoxin reductase, which is involved in protection against oxidative stress (52). Importantly, measuring the overall redox status of endoplasmic reticulum during protein folding and secretion by detection of H_2_O_2_ or GSH/GSSG is counterfactual (53). For example, in *Caenorhabditis elegans*, the GSH couple are not the main redox buffers (54)_,_ demonstrating that a probe to detect the overall redox status is extremely useful, as has been suggested previously by roGFP reporter studies (55). Nevertheless, defining the specificity of a probe toward a certain important oxidative molecule would be highly advantageous. Therefore, further optimization of pHaROS for desired specificity, such as that of the HyPer probe for measuring H_2_O_2_ or Grx1-roGFP2 for reporting GSH/GSSG ratio, could extend its great utility in studying a particular signaling pathway. Here, we also demonstrate that a specific probe for GSSG could be generated by simply modifying the pHaROS sensor.

Because the 561/488 ratio is pH-dependent, and moreover is correlated with pH values in the physiological range, the fluorescent properties of mBeRFP within pHaROS enable real-time imaging of cellular pH. Furthermore, the p*K_a_* value of mBeRFP protein is 5.8, which is highly suitable for visualization of both cytosolic and nuclear pH of the yeast cell (56) (Figs. 4 and 5). In our study, we also show that the pH range of pHaROS can be expanded by replacing mBeRFP with a different pH probe such as pHred, to meet specific requirements for different organelles and cells. Although pHred has relatively weak fluorescence in yeast, pHaROS-red was able to display its pH sensitivity in the alkaline range. Therefore, pHaROS and its variants are the ideal option for simultaneously measuring cellular pH and Eh also at a wide range of pH values.

pHaROS is a powerful tool for shedding light on the cross-talk between pH and redox conditions. One of the great advantages of the pHaROS sensor over conventional fluorescent probes is the simultaneous detection of both intracellular pH and redox state, which could indicate the differences between pH and redox changes upon the perturbations of cellular homeostasis. The pH and redox probes that are currently in widespread use are all based on GFP, which can lead to complications when pH and redox states are monitored simultaneously. To date, it has been almost impossible to put both a pH probe and a redox sensor into a single organism due to the spectral overlap of the available probes. Our results suggest that iLOV protein could be used as a redox probe and combined with the pH probe mBeRFP as components of the pHaROS sensor. Tests in *S. cerevisiae* showed a clear shift in iLOV fluorescence in response to the redox state, accompanied by mBeRFP fluorescence changes according to the pH state. Both Eh and pH states, reflecting electron transmission and proton transfer, respectively, are crucial parameters of the cell metabolism. Indeed, imaging of yeast mutant *gpx3*Δ cells using pHaROS revealed a higher oxidative level in *gpx3*Δ than that in the WT BY4741 cells.

pHaROS imaging can be used to distinguish the subtle effects of certain regulators of redox or pH homeostasis in cells. For example, we showed that the oxidant H_2_O_2_ alters the overall redox potential of the cell when applied in sufficiently high concentrations. However, we also found that H_2_O_2_ reduced the cytosolic pH of yeast cells, which is consistent with the previous observations that 1 mm H_2_O_2_ induced a drop in pH of ∼1 unit in yeast cells (6, 57). Therefore, changes in both redox and pH must be considered when evaluating the effects of H_2_O_2_ on cellular redox homeostasis. It is well-known that pH affects protein conformation, enzymatic activity, and signal transduction, and that protons are the product of many chemical reactions. These previous observations should be re-evaluated using pHaROS, which is perfectly suited to study the cross-talk between redox and pH intracellular signaling processes.

Interestingly, we observed oscillations of both pH and redox potential in the nucleus during the yeast budding. The first increase of the nuclear redox status accompanies the DNA replication during the S phase, and the second occurs before the nucleus migration. The pH peak in mother cell nucleus during budding is in the middle of the S phase. We observed changes in the GSH/GSSG redox couple in yeast cells during budding using a NLS-Grx1-pHaROS. Although the GSH/GSSG ratio is lower in *glr1*Δ mutant cells than in BY4741 cells, it shows similar changes in both types of cells during budding, suggesting that the GSH/GSSG redox couple contribute to the oscillation of the redox potential, which is required for budding. We assume that the regulation of pH and redox homeostasis in the nucleus is essential for the activity of several enzymes, and is likely to affect DNA replication (58) through histone acetylation of H3 (59–61) and phosphorylation of the cell cycle factors (62). Fluctuations of nuclear pH and redox state are mirrored by changes in DNA synthesis and compaction, chromosome segregation, and cell division. Probably, some intertwined cytosolic and nuclear pH and redox homeostasis may optimize cell proliferation and mitosis in yeast. It is clearly important to regulate pH and redox homeostasis in the nucleus of yeast cells due to the cross-talk between these signaling pathways. To find the important genes that modulate the interactive effect of pH and redox potential during yeast cell cycle genetically, a screening system based on pHaROS could be established. Further outcomes of these and other pHaROS-based studies would help to reveal the underlying processes and the mechanism of pH and redox oscillation during the cell cycle.

## Experimental procedures

### Materials

Aldrithiol, lipoate, dihydrolipoate, hydrogen peroxide, oxidized and reduced GSH, menadione, xanthine, and xanthine oxidase were purchased from Sigma. LOV, iLOV, and LOV2.1 amino acid sequences were generously supplied by John M. Christie's laboratory. pRSET-mBeRFP plasmid was provided by the Wuhan National Laboratory for Optoelectronics. INVSc1 yeast cell were obtained from Invitrogen. WT yeast cell BY4741, *gpx3*Δ, and *glr1*Δ were purchased from Euroscarf.

### Genetic constructs

iLOV was cloned into pYES2 (Invitrogen) using HindIII and BamHI restriction sites, then mBeRFP was cloned into pYES2-iLOV using the EcoRI and XhoI sites to allow inducible expression in yeast cells; the bases between the two enzyme-cleavage sites produced a short amino acid linker sequence, GSTSNGRQCAGIL according to pYES2 vector, between iLOV and mBeRFP. The length of the polypeptide linker was designed following the previous study (63). To merge other genes, such as SU9, we placed a point-mutation in BamHI by using the QuikChange Lightning Site-directed Mutagenesis Kit from Agilent Technologies. For a constant expression of pHaROS protein, we replaced pGAL with a constant promoter pACT1 in pYES2-iLOV-mBeRFP. iLOV and pHred sequences were cloned into pYES2 plasmid by homologous recombination sequentially and generated another probe named pHaROS-red. GRX1 sequence was combined with the pHaROS probe by using the same homologous recombination method to generate the GRX1-pHaROS probe. In addition, the pHaROS fragment was cloned into mammalian plasmid pcDNA3.1 and transfected into the U87 cell line for the use of pHaROS probe in mammalian cells. A GSH/GSSG-specific probe targeted to the nucleus was constructed by using two tandem SV40 NLS tag PPKKKRKV, which were synthesized and linked to N-terminal of GRX1-pHaROS and Grx1-roGFP2 sequences, respectively. An extra SV40 NLS tag PPKKKRKV was synthesized and linked to C-terminal of Grx1-roGFP2 to force Grx1-roGFP2 protein stay in the nucleus.

### Protein expression and purification

pGEX-iLOV plasmids were transformed into *E. coli* strain JM109, which were incubated at 37 °C with 220 rpm agitation overnight. iLOV protein expression was induced by adding 0.4 mm isopropyl β-d-1-thiogalactopyranoside to a culture grown at 16 °C and 180 rpm when *A*_600_ reached 0.4. iLOV protein purification was carried out as described previously (17). In addition, the pRSET-mBeRFP plasmid was transformed into *E. coli* strain BL21. The recombinant BL21 bacteria were cultured overnight at 37 °C and 220 rpm until the *A*_600_ reached 0.8, then ampicillin was added (final concentration of 100 μg/ml) and bacteria were incubated for an additional 24 h at 37 °C and 220 rpm. Cells were pelleted by centrifuging at 8000 rpm, and resuspended in 30 ml of lysis buffer (50 mm Tris-HCl, pH 8.0, 250 mm NaCl) containing 1 mm phenylmethylsulfonyl fluoride. Bacteria were lysed using an ultrasonic probe (Branson 102c) on ice by three bursts of 10 min each. The cell lysate was clarified by centrifugation at 12,000 rpm for 1 h. The iLOV and mBeRFP protein was purified on a Ni^+^ column, in accordance with the manufacturer's protocols.

### FRET assay

The fluorescence lifetime of iLOV in the absence and presence of mBeRFP moiety was measured using a home-built fluorescence lifetime setup (Harp300, Picoquant) coupled with the time-correlated single-photon counting (TCSPC) module (time resolution < 4 ps) and a broadband tunable pulse femtosecond Ti:Sappihire laser (Chameleon Ultra II, Coherent Inc.). The 457 nm femtosecond excitation source was obtained from the secondary harmonic generation with BBO nonlinear crystal to excite the iLOV protein, and the repetition rate was regulated down to 40 MHz with a pulse selection system (Conoptics, model 305). The emission passed through a band-pass filter (ET540/30m, Chroma) and was detected by a micro photodevice (Picoquant) with the time resolution of less than 40 ps. The emission lifetime was determined by fitting the emission trajectory with single exponential decay function.

### Titration of iLOV proteins

After GST-iLOV protein was purified, it was digested with protease trypsin, and the iLOV proteins were recovered. To test the maximum redox responses of the novel probe, the iLOV proteins were treated with 50 mm DTT and illuminated for 5 min with a 450-nm blue LED to form the fully reduced iLOV. Alternatively, iLOV was treated with 10 mm H_2_O_2_ for 30 min to induce its full oxidation. iLOV was titrated using GSH/GSSG buffer at concentrations varying from 0:10 to 10:0 mm in 1 mm increments; dihydrolipoate and lipoate were also used in titration. The purified iLOV protein was diluted to 1 μm in MES/HEPES/Tris buffer of various pH values, and incubated for 1 h, before the fluorescence spectrum was examined in a Hitachi F4500 fluorescence spectrometer.

### In vitro pH calibration

*In vitro* pH calibration was performed in 50 mm MES/HEPES/Tris calibration buffer, with 1 m NaOH and 1 m HCl being used to adjust the buffer to different pH values ranging from 5.0 to 9.0. mBeRFP protein was diluted to a final concentration of 1 μm into 1000 μl of calibration buffer. The fluorescence spectrum and fluorescent intensity changes were detected using a Hitachi F-4500 fluorescence spectrophotometer. The instrument parameters were set as follows: excitation wavelength, 450 nm; emission (EM) range, 470–600 nm; scan speed, 1200 nm/min; delay, 1 s; excitation slit, 5 nm; emission slit, 10 nm; photomultiplier tube (PMT) voltage, 700 V; and response, 2.0 s.

### In vitro Eh calibration

*In vitro* Eh calibration was carried out in 50 mm DTT solution at pH 8.2. Oxidized DTT was used to adjust the redox potential between −192 and −334 mV. iLOV protein was diluted to a final concentration of 1 μm in 1000 μl of calibration buffer. The fluorescence spectrum and fluorescent intensity changes were detected using a Hitachi F-4500 fluorescence spectrophotometer. Instrument parameters were set as follows: emission wavelength, 510.0 nm; excitation range, 300–500 nm; scan speed, 1200 nm/min; delay, 1 s; excitation slit, 5.0 nm; emission slit, 10.0 nm; PMT voltage, 700 V; and response, 2.0 s.

### Preparation of transgenic S. cerevisiae

pYES2-iLOV-mBeRFP plasmid was transferred into *S. cerevisiae* strain INVSc1 by electroporation. Five milliliters of the transgenic INVSc1 strain was grown in SC-U medium overnight at 30 °C with shaking, and an aliquot of the overnight culture was diluted to obtain an *A*_600_ of 0.4 in 5 ml of induction medium. Cells were induced by overnight growth in induction medium (2% raffinose and 2% galactose) at 30 °C and 220 rpm, followed by shaking for another 10 h.

### Confocal laser scanning microscopy manipulation procedure

Microscope slides (24 × 32 and 0.13–0.17 mm thick) or glass-bottomed Petri dishes were first rinsed with 80% ethanol and then rinsed in reverse osmosis water several times. Approximately 30–50 μl of 0.1 mg/ml of concanavalin A (ConA) solution was spotted onto the cleaned slides, which were then dried at 30 °C overnight. 30 μl of yeast cell suspension was placed on the area coated with ConA and incubated for 10 min at 30 °C, after which time the excess cells were rinsed off and the culture medium was added to the adhered cells.

### Imaging setup for living yeast

We used a Zeiss LSM 710 confocal microscope with lasers at 405, 488, and 561 nm (objective: DIC III, ×63, 1.4 NA (oil); detector, PMT; immersion oil, Immersol^TM^ 518F (ne = 1.518, ve = 45)). The imaging settings were as follows: laser 1, 488 nm, 6%; laser 2, 561 nm, 6%; pinhole: 1.97 airy units; gain, 780. The track settings were as follows: track 561 nm, channel mBeRFP, emission range from 595 to 630 nm; track 488 nm, channel iLOV emission range from 495 to 530 nm, channel mBeRFP emission range from 595 to 630 nm. Track ratio was ratio 1, track 561 channel mBeRFP/track 488 channel mBeRFP. Acquisition settings were: scan model: frame, frame size: 512 × 512; line step, 1; speed, 9; averaging number, 4; bit depth, 16 bit; mode, line; direction, double direction; method, mean; and zoom, 4. First, the microscope, mercury lamp, and laser controller were switched on and the software was started. Imaging setting was then applied to the software. A ×63 oil immersion (NA = 0.45) objective was used for imaging. Because of the *in vivo* reversible photobleaching property of iLOV protein (21, 38), an interval of 2 or 5 min was set during experimental conditions for scanning, which resulted in enough stability to collect the fluorescence information of images.

### In vivo pH and Eh calibration of yeast strains with pHaROS probe

The transgenic yeasts were induced by galactose to express the pHaROS fusion protein *in vivo*. Log phase yeast cells were incubated at one of several examined pH buffers before being submitted to fluorescence imaging with a Zeiss 710 confocal microscope. To keep the intra- and intercellular pH identical, yeast cells were pretreated with 0.1% digitonin for 10 min. Yeast cells expressing pHaROS were diluted into calibrated pH buffer to ∼1 × 10^4^ cells/ml concentration. Cells adhered to the ConA-covered glass slides were imaged using the above parameters. The procedure for the *in vivo* Eh calibration was the same as that used for *in vivo* pH calibration, except using the Eh calibration buffer instead of the pH calibration buffer.

### Time course observation of GRX1-pHaROS probe in yeast cells

Yeast cells expressing GRX1-pHaROS were adhered to confocal a Petri dish by ConA. A Zeiss 710 microscope was used for fluorescence imaging. After 5 time points of imaging, GSSG was added to a Petri dish at different concentrations and fluorescence images were captured every 2 min.

### Spinning disc confocal settings for yeast imaging

Transgenic yeast strain BY4741 and *gpx3*Δ were used in the spinning disc confocal microscopy (ANDOR) experiments. The yeasts were cultured to reach the log phase and adhered to Petri dishes with ConA. The initial settings for the spin disc confocal microscopy were as follows: ×100 oil lens; frame size, 1024 × 1024; emission gain, 200; exposure time, 200 ms; bit depth, 16; excitation 1, 488 nm; excitation 2, 561; filter 1, 514/30 nm; and filter 2, 607/30 nm. To test the influence of different redox agents on the cells, images were captured every 2 min.

### High throughput imaging of the yeast cell during budding process

Transgenic BY4741 and *glr1*Δ cells expressing NLS-Grx1-roGFP2 and NLS-Grx1-pHaROS were cultured to pre-log phase and treated with α-factor for 180 min, then washed to remove α-factor and seeded into a 96-well Cell carrier plate (PerkinElmer). A Operetta High Content Screening (HCS) instrument (PerkinElmer) was used to capture fluorescence images for 6 h with 5-min intervals.

### Yeast budding assay

WT yeast strain BY4741 was used in the budding assay. Log phase BY4741 cells were imaged by spinning disc confocal microscopy at 30 °C overnight, and images were captured every 5 min. The pHaROS fluorescence intensity of each channel was calculated using ImageJ software.

### Imaging and data analysis

Because iLOV protein has a single excitation spectrum, it is difficult to generate a fluorescence ratiometric image of the redox status. As the fluorescence of mBeRFP fluctuates according to the pH, it is not possible to generate the Eh ratio directly from iLOV fluorescence. However, as shown in Fig. 3*E*, a fitting curve was obtained based on pH and mBeRFP fluorescence ratio 561 nm/488 nm (R561/R488) according to the Hill equation.

(Eq. 1) $${\text{Ratio}}_{\text{R}561/\text{R}488}={\text{ratio}}_{\min}+\left({\text{ratio}}_{\max}-{\text{ratio}}_{\min}\right)/\left[1+{\left(\text{p}K_{a}/\text{pH}\right)}^{n}\right]$$

Another fitting curve based on pH and mBeRFP fluorescence intensity (*I*) of excitation 561 nm was obtained according to the following equation.

(Eq. 2) $$I_{\text{R}561}=I_{\min}+\left(I_{\max}-I_{\min}\right)/\left[1+{\left(\text{p}K_{a}/\text{pH}\right)}^{n}\right]$$

R561 max was calculated according to Equation 2. Because the ratio of *I*_max_/*I*_min_ is constant under the same experimental conditions, *I*_min_ = m*I*_max_.

(Eq. 3) $$I_{\max}=I_{\text{R}561}\left[1+{\left(\text{p}K_{a}/\text{pH}\right)}^{n}\right]/\left[1+\text{m}{\left(\text{p}K_{a}/\text{pH}\right)}^{n}\right]$$

The relationship of iLOV fluorescence intensity and Eh was detected and plotted in Fig. 3*G*, whereby the fitting curve was generated by the formula (Equation 4) as follows.

(Eq. 4) $$I_{\text{G}488}=\text{a}+\text{b}\times \text{Eh}$$

Using the max value of mBeRFP fluorescence intensity as a constant to generate the ratio value of Eh in Fig. 3*H*, the following equations can be used.

(Eq. 5) $${\text{Ratio}}_{\text{G}488/\text{R}561\text{M}}=I_{\text{G}488}/I_{\max}$$

(Eq. 6) $${\text{Ratio}}_{\text{G}488/\text{R}561\text{M}}=I_{\text{G}488}\left[1+\text{m}{\left(\text{p}K_{a}/\text{pH}\right)}^{n}\right]/I_{\text{R}561}\left[1+{\left(\text{p}K_{a}/\text{pH}\right)}^{n}\right]$$

There are two ways to obtain *R*_561 nm_ max: one is to use digitonin and pH 8.2 Tris buffer to generate *R*_561 nm_ max image of yeast cells; the other is to calculate *R*_561 nm_ max based on the fitting curve of R561 and pH. We used both when generating the ratio images.

## Author contributions

H. Z. and Y. Z. methodology; H. Z. and C.-P. S. writing-original draft; H. Z., Y. Z., L. B., X. Z., and C.-P. S. writing-review and editing; Y. Z. conceptualization; Y. Z. formal analysis; M. P., Y. S., and Y. H. data curation; Y. M. resources; X. Z. and C.-P. S. supervision; C.-P. S. funding acquisition; C.-P. S. project administration.

## Supplementary Material

Supporting Information

## Footnotes

3 The abbreviations used are:

LOV: light, oxygen, or voltage-sensing
pHaROS: p H- and redox-sensitive fluorescent protein
PMT: photomultiplier tube
ConA: concanavalin A
Eh: electric potential
NLS: nuclear localization sequence.
