# Supplementary material for: Dynamic imaging of cellular pH and redox homeostasis with a genetically encoded dual-functional biosensor, pHaROS, in yeast
Source: J Biol Chem. 2019 Sep 5;294(43):15768–80. doi: 10.1074/jbc.RA119.007557 (PMC6816096; doi:10.1074/jbc.RA119.007557)
Supplement: Supporting Information [file supp_294_43_15768__index.html]

Dynamic imaging of cellular pH and redox homeostasis with a genetically encoded dual-functional biosensor, pHaROS, in yeast — A dual-functional probe for pH and redox potential — Dynamic imaging of cellular pH and redox homeostasis with a genetically encoded dual-functional biosensor, pHaROS, in yeast — A dual-functional probe for pH and redox potential — Supporting Information 

# Dynamic imaging of cellular pH and redox homeostasis with a genetically encoded dual-functional biosensor, pHaROS, in yeast

## Supporting Information

- Supporting Information - Supplementary figures plus legends
- Supplementary video 1 - Example video of pH ratio change during budding process (5 min interval).
- Supplementary video 2 - Example video of Eh ratio change during budding process (5 min interval).
- Supplementary video 3 - Example video of pH ratio change of yeast that cannot enter M phase (5 min interval).
- Supplementary video 4 - Example video of Eh ratio change of yeast that can not enter M phase (5 min interval).
- Supplementary video 5 - Example video of pH ratio change in yeast cell that cannot enter S phase (5 min interval).
- Supplementary video 6 - Example video of Eh ratio change in yeast cell that cannot enter S phase (5 min interval).
- Supplementary video 7 - Example video of pH ratio change in yeast cell that cannot finish mitosis during budding. (5 min interval).
- Supplementary video 8 - Example video of Eh ratio change in yeast cell that cannot finish mitosis during budding. (5 min interval).
