# Supplementary material for: Dynamic imaging of cellular pH and redox homeostasis with a genetically encoded dual-functional biosensor, pHaROS, in yeast
Source: J Biol Chem. 2019 Sep 5;294(43):15768–80. doi: 10.1074/jbc.RA119.007557 (PMC6816096; doi:10.1074/jbc.RA119.007557)
Supplement: Supporting Information [file supp_RA119.007557_143112_2_supp_388154_px9b4v.pdf]

Dynamic imaging of cellular pH and redox homeostasis using a genetically encoded dual-functional biosensor pHaROS in yeast

Hang Zhao[#], Yu Zhang[#], Mingming Pan, Yichen Song, Ling Bai, Yuchen Miao, Yanqin Huang, Xiaohong Zhu, and Chun-Peng Song*

Supporting Information

List of materials:

Supplementary Figure S1. Fluorescent characteristics of LOV proteins in *E.coli*.

Supplementary Figure S2. Titration analysis of the iLOV protein with the GSH/GSSG redox couple.

Supplementary Figure S3. Redox characteristics of iLOV protein in different redox couples and fluorescent properties of iLOV in yeast.

Supplementary Figure S4. iLOV protein is insensitive to pH and divalent cations.

Supplementary Figure S5. iLOV protein in *S. cerevisiae* cell is insensitive to copper ion.

Supplementary Figure S6. mBeRFP protein is insensitive to redox agents and divalent ions.

Supplementary Figure S7. Fluorescence decay of iLOV in the absence (black) and in the presence of mBeRFP partner (pHaROS, red).

Supplementary Figure S8. pHaROS is capable of detecting changes in pH and redox potential.

Supplementary Figure S9. Construction and fluorescence characterization of two tailored pHaROS variants, GRX1-pHaROS and pHaROS-red.

Supplementary Figure S10. Redox potential and pH changes upon an addition of H_2O_2 in U87 cells expressing pHaROS probe.

Supplementary Figure S11. Effect of redox agents on pH of yeast cells.

Supplementary Figure S12. Yeast cell size is affected by cellular redox state.

Supplementary Figure S13. Incomplete budding process in WT yeast cells companied with abnormal pH or Eh changes.

Supplementary Figure S14. Recording of the redox changes by Grx1-roGFP2 and GRX1-pHaROS probes in the nucleus of yeast strain BY4741 and *glr1Δ* during budding.

Supplementary video 1. Example video of pH ratio change during budding process (5 min interval).

Supplementary video 2. Example video of Eh ratio change during budding process (5 min interval).

Supplementary video 3. Example video of pH ratio change of yeast that cannot enter M phase (5 min interval).

Supplementary video 4. Example video of Eh ratio change of yeast that cannot enter M phase (5 min interval).

Supplementary video 5. Example video of pH ratio change in yeast cell that cannot enter S phase (5 min interval).

Supplementary video 6. Example video of Eh ratio change in yeast cell that cannot enter S phase (5 min interval).

Supplementary video 7. Example video of pH ratio change in yeast cell that cannot finish mitosis during budding. (5 min interval).

Supplementary video 8. Example video of Eh ratio change in yeast cell that cannot finish mitosis during budding. (5 min interval).

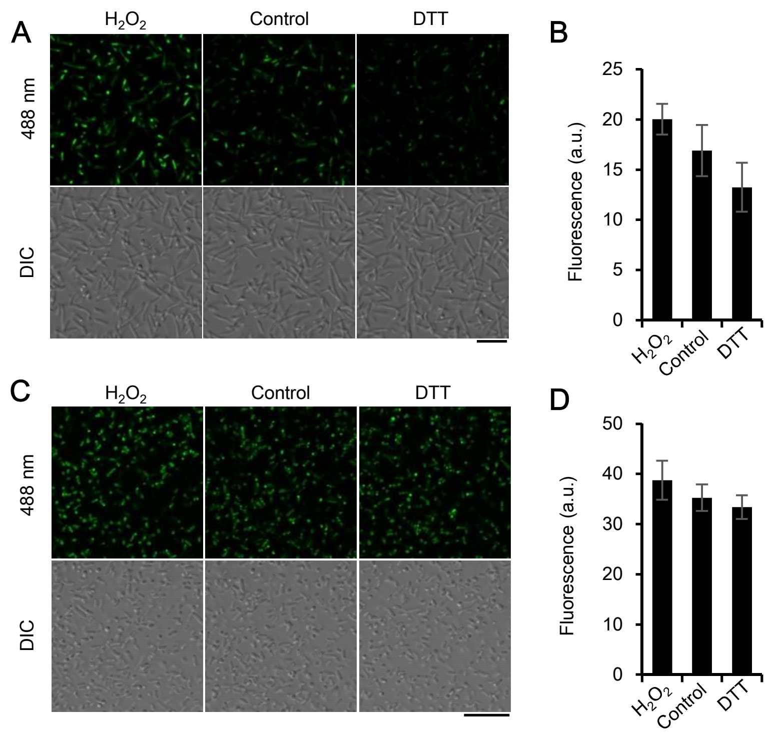

Supplementary Figure S1. Fluorescent characteristics of LOV proteins in *E.coli*. (A) iLOV protein was expressed in *E. coli* and treated with 2 mM H_2O_2 or 5 mM DTT. **(B)** The change in fluorescence intensity of iLOV in A. **(C)** LOV2.1 protein was expressed in *E. coli* and treated with 2 mM H_2O_2 or 5 mM DTT. **(D)** The fluorescence intensity change of LOV2.1 in panel C. All of the measurements were performed at least in triplicate. Bar = 10 μm.

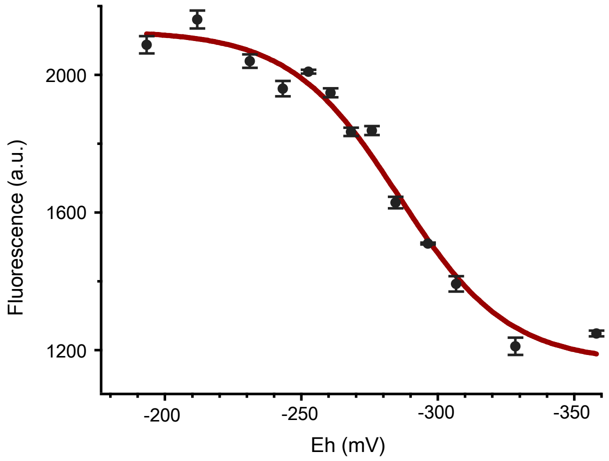

Supplementary Figure S2. Titration analysis of the iLOV protein with the GSH/GSSG redox couple. The fluorescence of iLOV (1 μM) was measured in titration buffer (pH 7.0, 25°C) containing different ratios of GSH/GSSG (10 mM total) corresponding to the designed redox potential (Eh) gradient. The excitation wavelength is 488 nm. a.u.: arbitrary units.

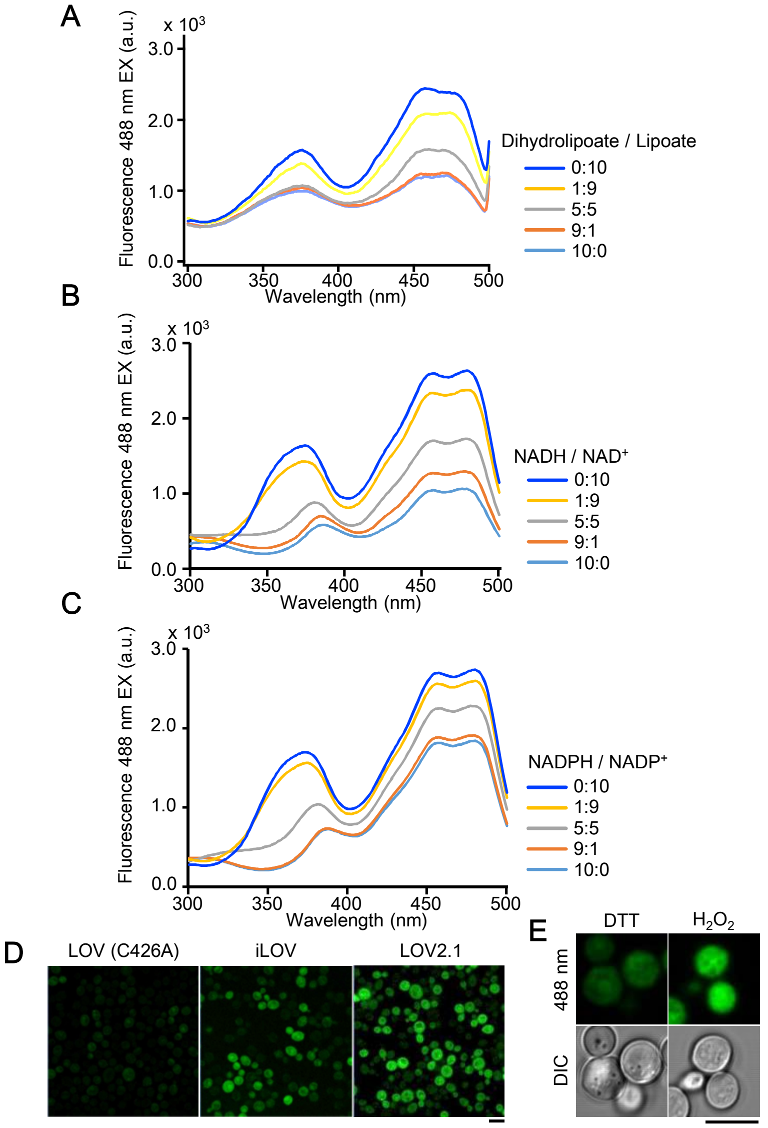

Supplementary Figure S3. Redox characteristics of iLOV protein in different redox couples and fluorescent properties of iLOV in yeast. (A) Titration analysis of iLOV with lipoate/dihydrolipoate buffer (10 mM). The experimental conditions were kept the same as for Figure 1e, except that the lipoate/dihydrolipoate redox couple was used. **(B)** Titration analysis of iLOV with different ratio of NAD^+/NADH buffer (1 mM total) as displayed by legends. **(C)** Titration analysis of iLOV with different ratio of $NADP^+$/NADPH buffer (1 mM total) as displayed. The excitation wavelength ranged from 300 to 500 nm in both cases (B and C). a.u.: arbitrary units. **(D)** The fluorescence intensities of the three LOV mutant proteins in yeast strain INVSc1. **(E)** Effect of Oxidation and reduction on addition of 5 mM DTT and 5 mM H_2O_2 to the *S. cerevisiae* strain expressing iLOV. Images were taken at an emission wavelength of 495-530 nm and excitation wavelengths of 488 nm. All of the measurements were performed at least in triplicate. Bar = 10 µm.

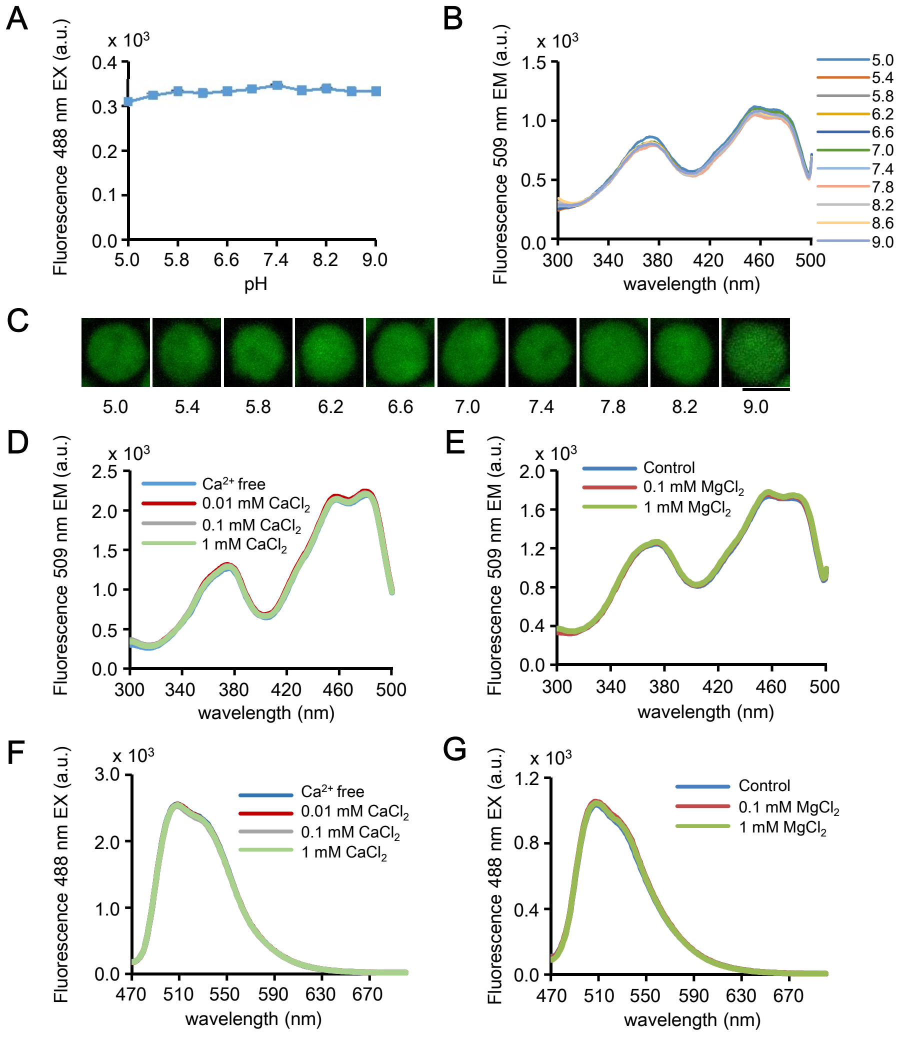

Supplementary Figure S4. iLOV protein is insensitive to pH and divalent cations. **(A)** The fluorescence intensity of iLOV protein was unaffected in the pH range of 5.0-9.0. **(B)** The excitation spectrum of iLOV protein in buffers of various pH. **(C)** *In vivo* imaging of iLOV in yeast in buffers of pH 5.0 to 9.0. Bar = 5 µm. **(D)** The excitation spectrum of iLOV in presence of 0.01 mM to 1 mM $CaCl_2$ solution. **(e)** The excitation spectrum of iLOV in presence 0.1 mM or 1 mM $MgCl_2$. **(F)** The emission spectrum of iLOV in presence of 0.01 mM to 1 mM $CaCl_2$ solution. **(G)** The emission spectrum of iLOV in presence of 0.1 mM or 1 mM $MgCl_2$ solution.

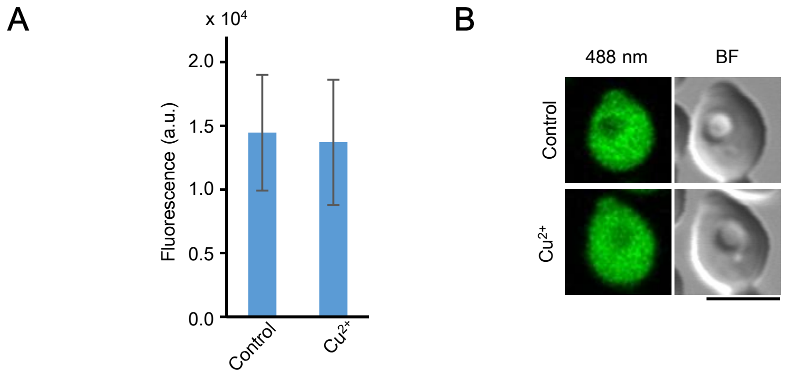

Supplementary Figure S5. iLOV protein in *S. cerevisiae* cell is insensitive to copper ion. (A) The fluorescence intensity of iLOV protein remains unaffected on treatment with 0.1 mM Cu^{2+}. **(B)** Effect of application of 0.1 mM Cu^{2+} on the fluorescence of the *S. cerevisiae* strain expressing iLOV. Bar = 5 μm.

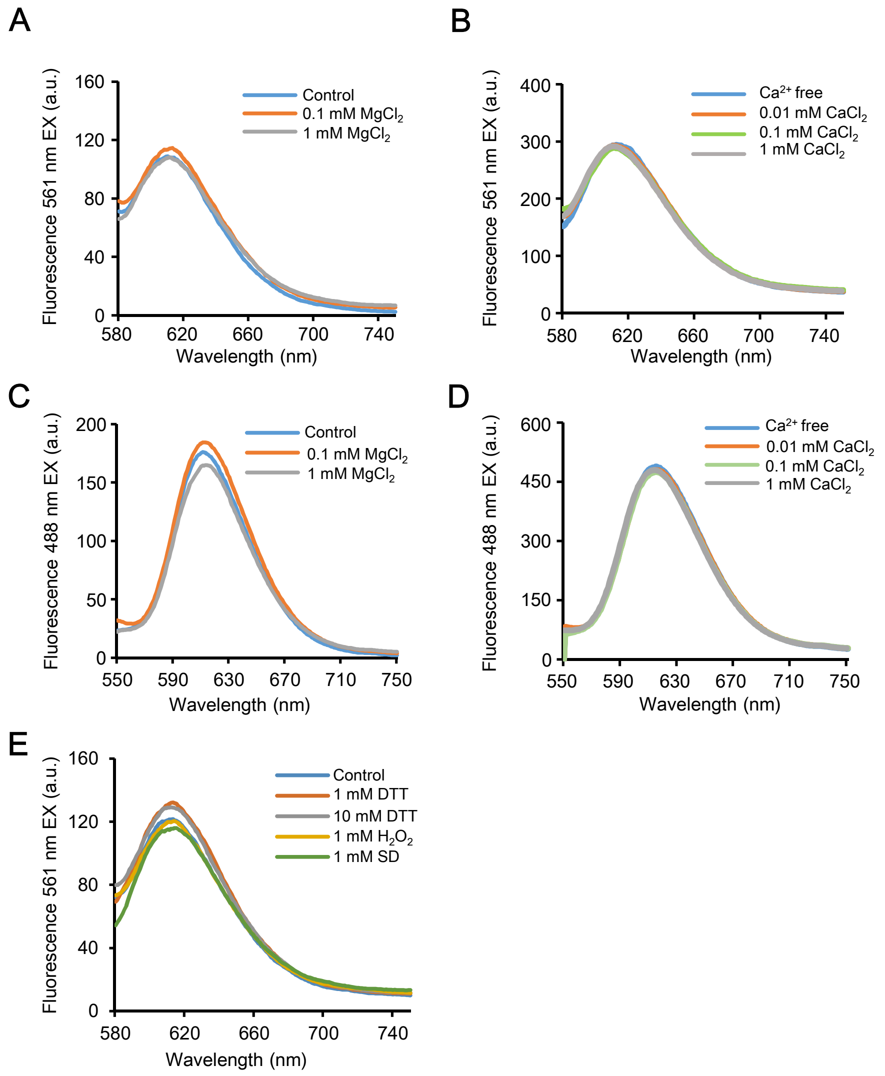

Supplementary Figure S6. mBeRFP protein is insensitive to redox agents and divalent ions. (A) The emission spectrum of mBeRFP in 0.1 mM or 1 mM $MgCl_2$ solution under 561 nm excitation. **(B)** The emission spectrum of mBeRFP in 0.01 mM to 1 mM $CaCl_2$ solution under 561 nm excitation. **(C)** The emission spectrum of mBeRFP in 0.1 mM or 1 mM $MgCl_2$ solution under 488 nm excitation. **(D)** The emission spectrum of mBeRFP in 0.01 mM, 0.1 mM or 1 mM $CaCl_2$ solution under 488 nm excitation. **(E)** The fluorescence intensity of mBeRFP protein is unaffected by redox solutions, here we used different concentration of DTT, H_2O_2 and sodium dithionite (SD) in pH 8.2 buffer.

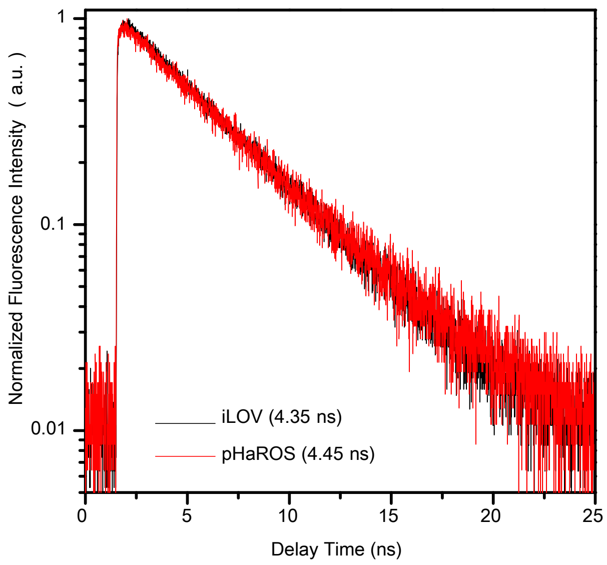

Supplementary Figure S7. Fluorescence decay of iLOV in the absence (black) and in the presence of mBeRFP partner (pHaROS, red). Purified iLOV and pHaROS protein was excited at 457 nm for a femtosecond by excitation source and the emission fluorescence was detected at 525-555 nm through a band-pass filter.

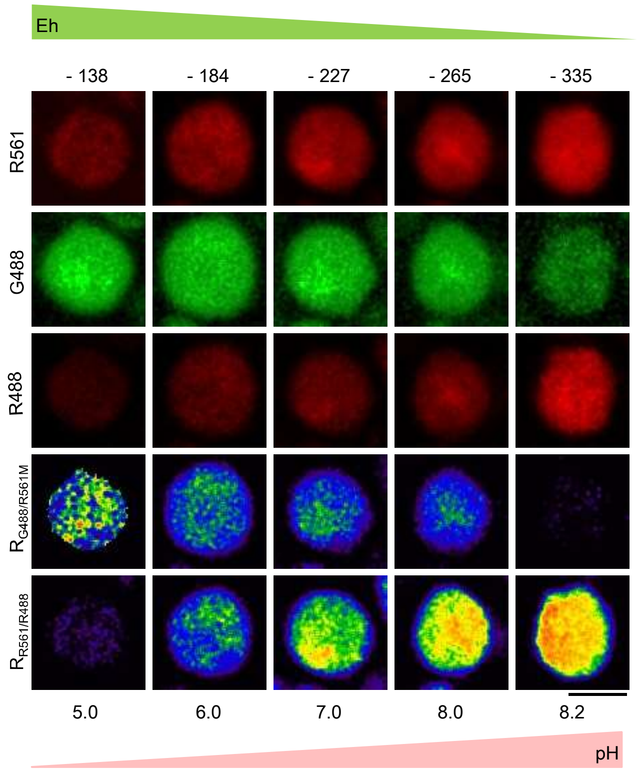

Supplementary Figure S8. pHaROS is capable of detecting changes in pH and redox potential. Confocal images of pHaROS in yeast in buffer at different pH/Eh (after digitonin treatment). Eh values of 50 mM DTT buffers of different pHs were detected by Thermo Orion Star with an ORP probe. Bar = 5 μm.

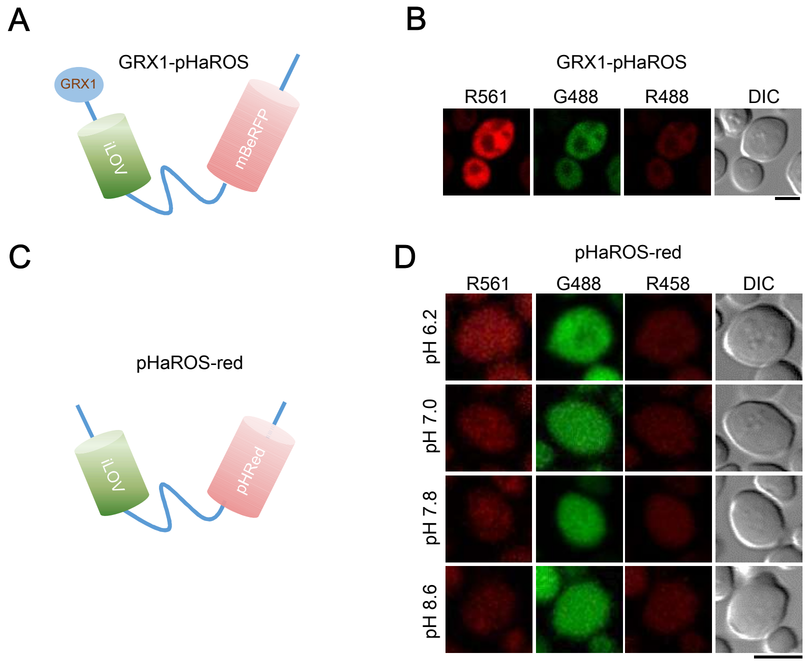

Supplementary Figure S9. Construction and fluorescence characterization of two tailored pHaROS variants, GRX1-pHaROS and pHaROS-red. (A) Schematic model of GRX1-pHaROS. GRX1 protein is linked at the N-terminal of pHaROS. **(B)** Fluorescence images of GRX1-pHaROS. **(C)** Schematic model of pHaROS-red. mBeRFP protein is substituted by another pH sensitive protein, pHred. **(D)** Fluorescence images of pHaROS-red in the buffer solutions with pH 6.2 - 8.6. Bar = 5 μm.

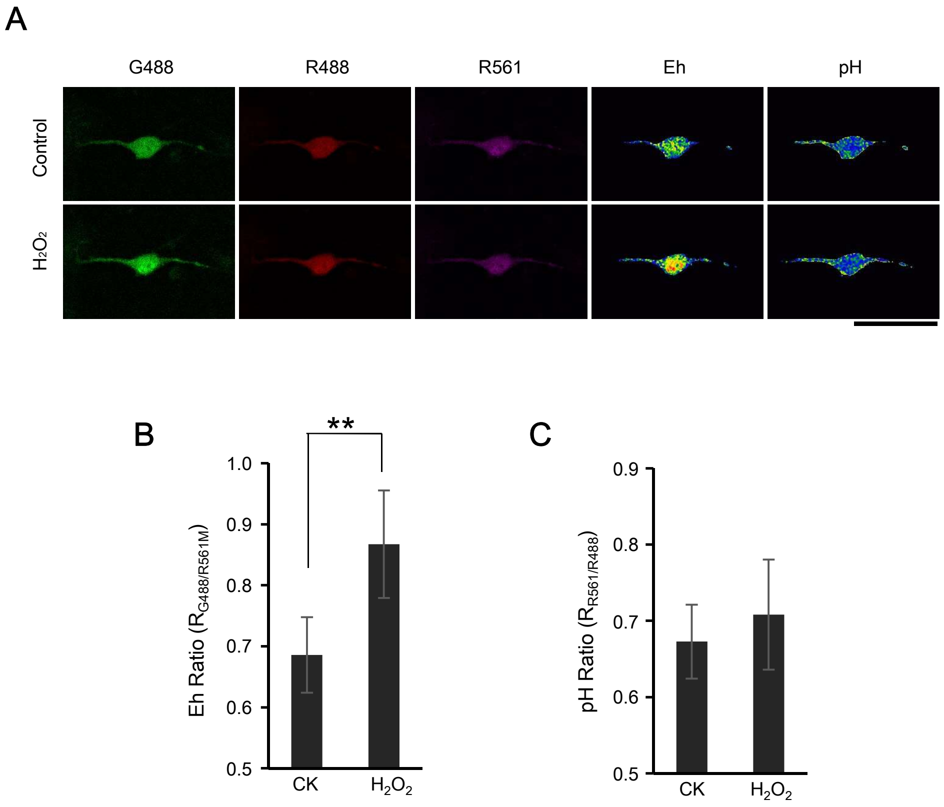

Supplementary Figure S10. Redox potential and pH changes upon an addition of H_2O_2 in U87 cells expressing pHaROS probe. (A) Transient transfection of pcDNA3.1-pHaROS plasmid into U87 cell. The images were acquired at 20 min after the 100 µM H_2O_2 treatment. Bar =100 µm. **(B)** Eh ratio changes in U87 cells treated by 100 µM H_2O_2 ($n > 18$). **(C)** pH ratio changes in U87 cells treated by 100 µM H_2O_2.

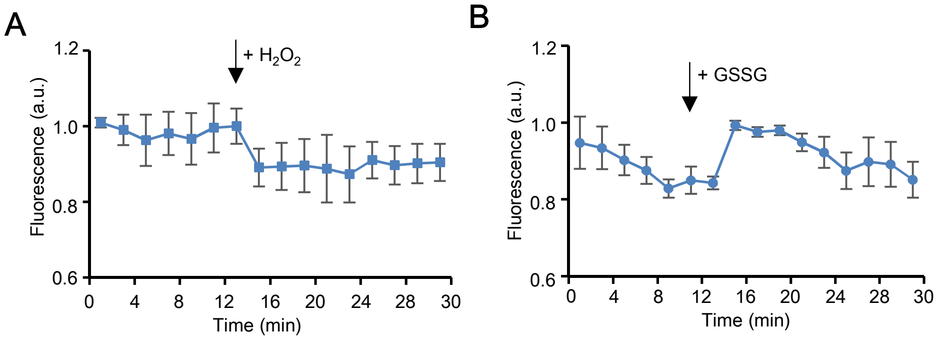

Supplementary Figure S11. Effect of redox agents on pH of yeast cells. **(A) and (B)** Yeast BY4741 were precultured with SNARF-1 pH probe for 1 h, then the SNARF-1 fluorescence was recorded every 2 min at the emission wavelength 580 - 630 nm when excited at 561 nm. 2 mM H_2O_2 and 2 mM GSSG were added after 10 mins (n = 12). The fluorescence intensity of SNARF-1 increases with the increase of environmental pH.

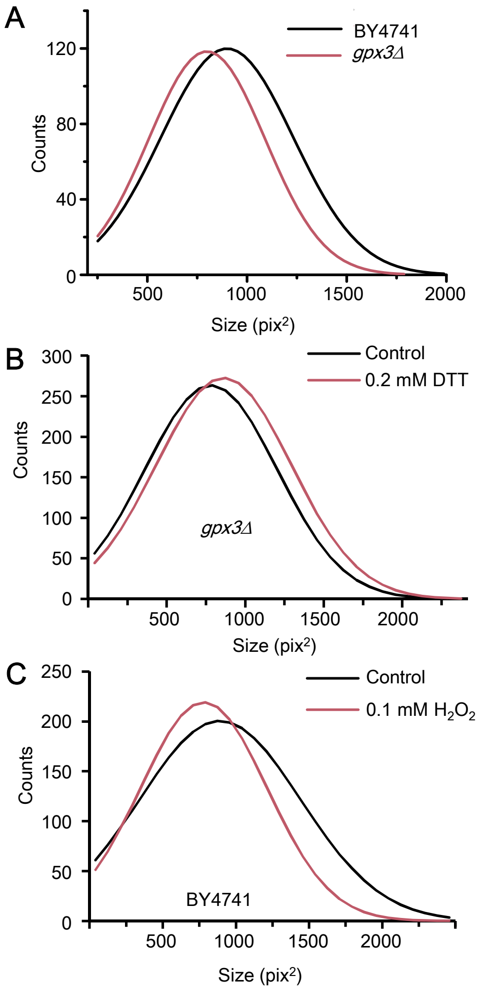

Supplementary Figure S12. Yeast cell size is affected by cellular redox state. **(A)** The population of mutant *gpx3Δ* yeasts is smaller than that of BY4741. **(B)** Population cell size of 0.2 mM DTT-treated *gpx3Δ* mutants is larger than that of DTT-untreated *gpx3Δ* yeast. **(C)** A decreased cell size of wild type yeast BY4741 after application of 0.1 mM H_2O_2.

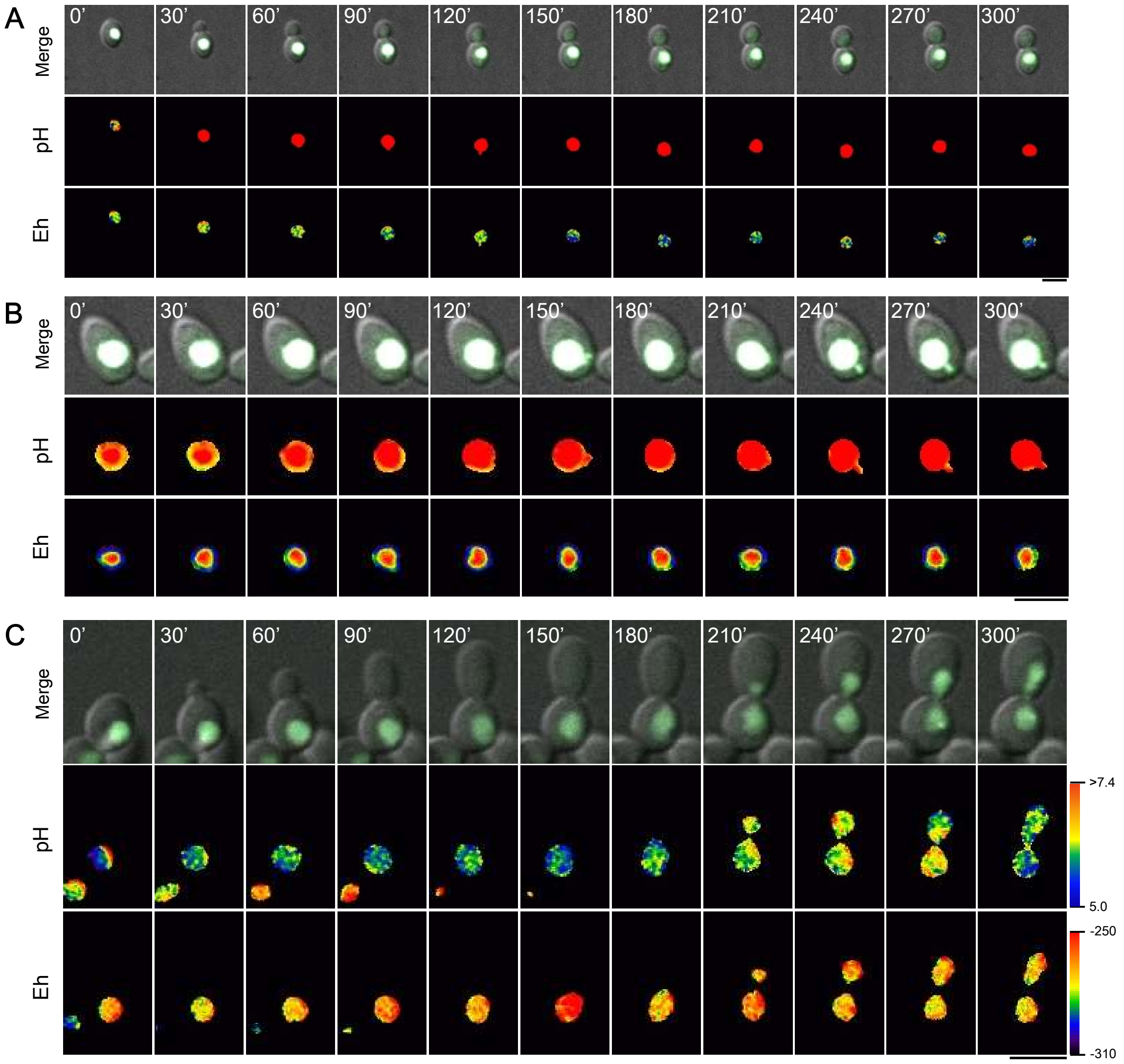

Supplementary Figure S13. Incomplete budding process in WT yeast cells companied with abnormal pH or Eh changes. (A) abnormal pH and Eh changes in yeast that are unable to enter the M phase (**Supplementary Videos 3** and **4**). **(B)** abnormal pH and Eh changes in yeast that are unable to enter the S phase. (**Supplementary Videos 5** and **6**). **(C)** abnormal pH and Eh changes in yeast that are unable to finish mitosis during sporulation. Fluorescence images were captured every 5 min and single cells were monitored for 5 h (**Supplementary Videos 7** and **8**). Bar = 5 μm.

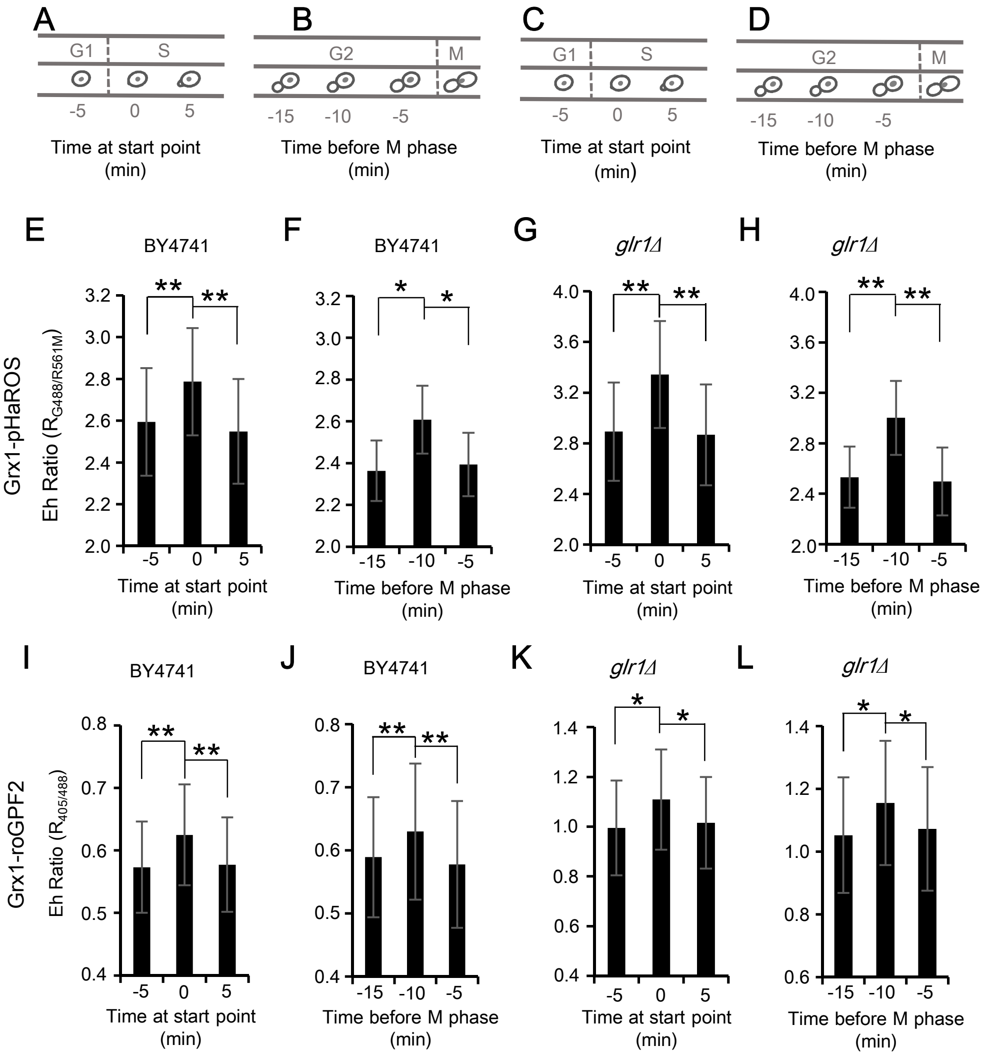

Supplementary Figure S14. Recording of the redox changes by Grx1-roGFP2 and Grx1-pHaROS probes in the nucleus of yeast strain BY4741 and *glr1Δ* during budding. The redox changes monitored by NLS-Grx1-pHaROS and NLS-Grx1-roGFP2 at start point of budding and before M phase of budding, respectively. Top panel – Schematic diagram of the cell stages during budding (A-D), which are correspondences to the middle and bottom panels; Middle panel – GSH/GSSG changes measured by NLS-Grx1-pHaROS during the cell cycle (E-H); Bottom panel – GSH/GSSG changes measured by NLS-Grx1-roGFP2 during the cell cycle (I-L). (** $p < 0.01$, * $p < 0.05$). Bars indicate the means ± s. d. ($n \geq 50$ yeast cells).

Supplementary video 1

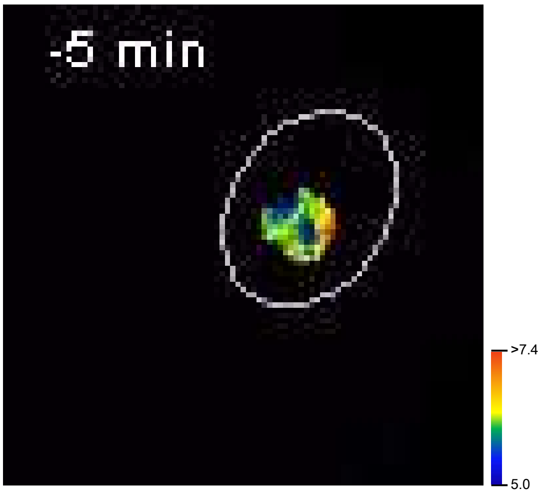

Example video of pH ratio change during budding process (5 min interval).

Supplementary video 2

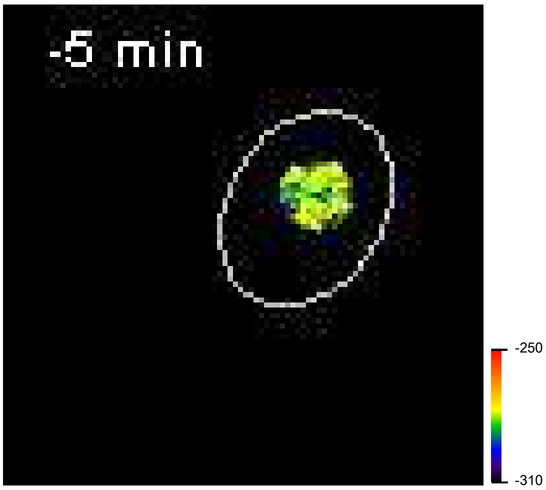

Example video of Eh ratio change during budding process (5 min interval).

Supplementary video 3

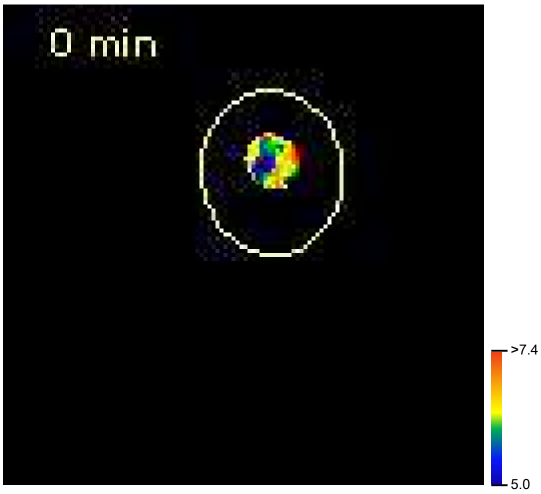

Example video of pH ratio change of yeast that cannot enter M phase (5 min interval).

Supplementary video 4

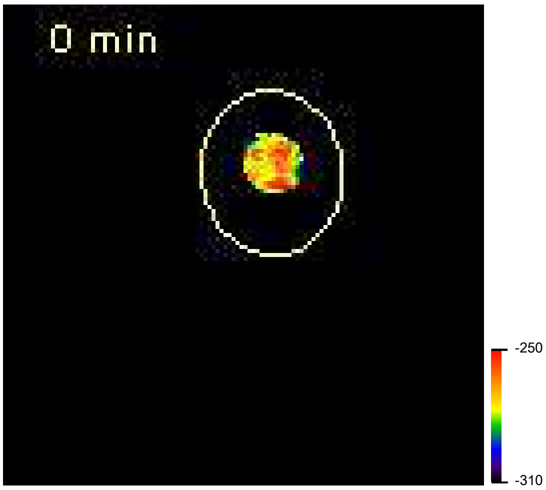

Example video of Eh ratio change of yeast that can not enter M phase (5 min interval).

Supplementary video 5

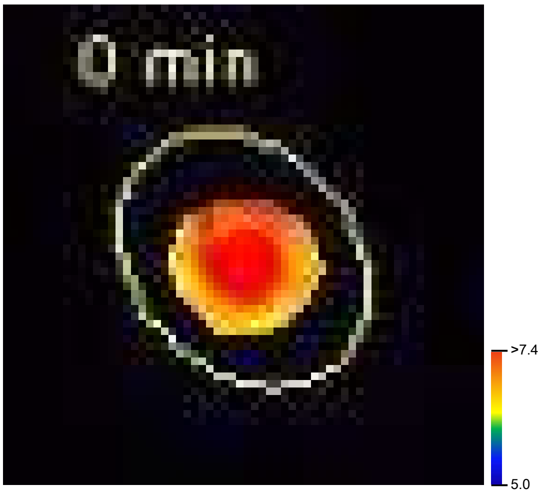

Example video of pH ratio change in yeast cell that cannot enter S phase (5 min interval).

Supplementary video 6

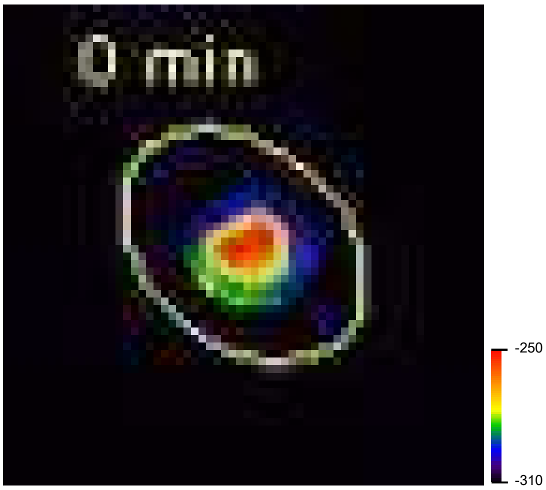

Example video of Eh ratio change in yeast cell that cannot enter S phase (5 min interval).

Supplementary video 7

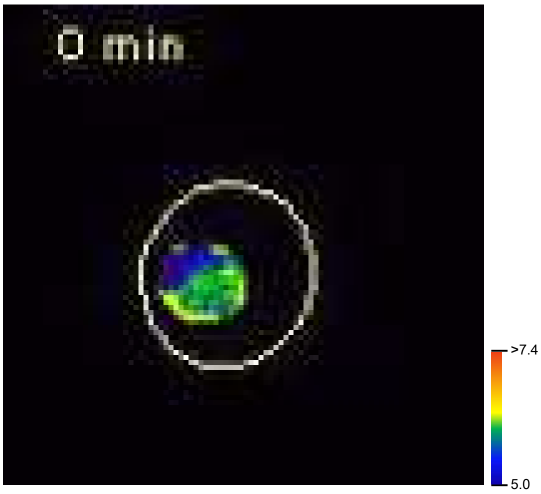

Example video of pH ratio change in yeast cell that cannot finish mitosis during budding. (5 min interval).

Supplementary video 8

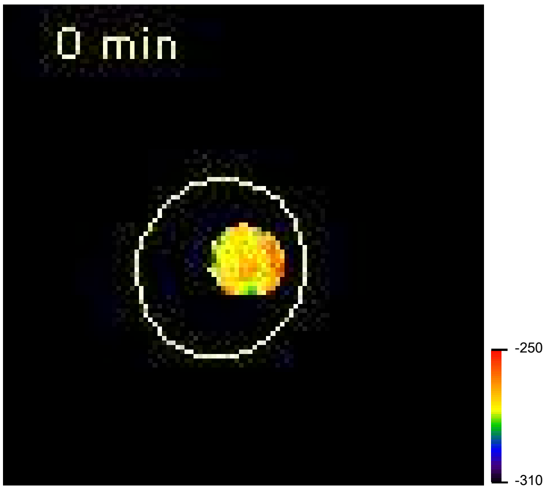

Example video of Eh ratio change in yeast cell that cannot finish mitosis during budding. (5 min interval).
